# RNA-Seq analysis of isolate- and growth phase-specific differences in the global transcriptomes of enteropathogenic *Escherichia coli* prototype isolates

**DOI:** 10.3389/fmicb.2015.00569

**Published:** 2015-06-12

**Authors:** Tracy H. Hazen, Sean C. Daugherty, Amol Shetty, Anup A. Mahurkar, Owen White, James B. Kaper, David A. Rasko

**Affiliations:** ^1^Institute for Genome Sciences, University of Maryland School of MedicineBaltimore, MD, USA; ^2^Department of Microbiology and Immunology, University of Maryland School of MedicineBaltimore, MD, USA

**Keywords:** enteropathogenic *Escherichia coli*, RNA-sequencing, diversity, regulation, pathogenesis

## Abstract

Enteropathogenic *Escherichia coli* (EPEC) are a leading cause of diarrheal illness among infants in developing countries. *E. coli* isolates classified as typical EPEC are identified by the presence of the locus of enterocyte effacement (LEE) and the bundle-forming pilus (BFP), and absence of the Shiga-toxin genes, while the atypical EPEC also encode LEE but do not encode BFP or Shiga-toxin. Comparative genomic analyses have demonstrated that EPEC isolates belong to diverse evolutionary lineages and possess lineage- and isolate-specific genomic content. To investigate whether this genomic diversity results in significant differences in global gene expression, we used an RNA sequencing (RNA-Seq) approach to characterize the global transcriptomes of the prototype typical EPEC isolates E2348/69, B171, C581-05, and the prototype atypical EPEC isolate E110019. The global transcriptomes were characterized during laboratory growth in two different media and three different growth phases, as well as during adherence of the EPEC isolates to human cells using *in vitro* tissue culture assays. Comparison of the global transcriptomes during these conditions was used to identify isolate- and growth phase-specific differences in EPEC gene expression. These analyses resulted in the identification of genes that encode proteins involved in survival and metabolism that were coordinately expressed with virulence factors. These findings demonstrate there are isolate- and growth phase-specific differences in the global transcriptomes of EPEC prototype isolates, and highlight the utility of comparative transcriptomics for identifying additional factors that are directly or indirectly involved in EPEC pathogenesis.

## Introduction

Enteropathogenic *Escherichia coli* (EPEC) have been associated with moderate to severe cases of diarrhea and are a leading cause of lethal diarrheal illness among young children in developing countries (Ochoa and Contreras, 2011; Kotloff et al., 2013). EPEC are identified by the presence of the locus of enterocyte effacement (LEE), which encodes a type III secretion system (T3SS) and the intimin adherence factor, which are involved in translocation of bacterial factors into host cells, and adherence to the surface of host cells. EPEC are also characterized by the absence of the Shiga-toxin genes, which is typically present in the enterohemorrhagic *E. coli* (EHEC) (Nataro and Kaper, 1998; Kaper et al., 2004). EPEC are further identified as typical EPEC by the presence of genes encoding the bundle-forming pilus (BFP), which is a type IV pilus typically carried by the EPEC adherence factor (EAF) plasmid. Meanwhile *E. coli* isolates that contain the LEE and do not carry the Shiga-toxin phage or the BFP genes are considered atypical EPEC (Nataro and Kaper, 1998). However, we have previously demonstrated that the atypical EPEC includes isolates with genomic similarity to typical EPEC and EHEC (Hazen et al., 2013).

The major components of EPEC pathogenesis that have been characterized to date are the T3SS encoded by the LEE region, additional T3SS effectors that are encoded by insertion elements or phages inserted within the genome, and the BFP (McDaniel et al., 1995; Nataro and Kaper, 1998; Kaper et al., 2004; Mellies et al., 2007; Nisa et al., 2013). Intimin, encoded by the *eae* gene, of the LEE region confers intimate attachment to host cells while the T3SS translocates effector proteins across the host cell membrane that result in the formation of the attaching and effacing lesions (Jerse et al., 1990; Donnenberg et al., 1993; McDaniel et al., 1995; McDaniel and Kaper, 1997; Garmendia et al., 2005). Once inside the host cell, the type III secreted effectors induce changes including rearrangement of the actin cytoskeleton to form pedestals that further facilitate adherence of EPEC to host cells (Sperandio et al., 1999; Wong et al., 2011; Clements et al., 2012; Nisa et al., 2013). The transcriptional regulation of the LEE and BFP regions have been extensively studied (Gomez-Duarte and Kaper, 1995; Puente et al., 1996; Mellies et al., 1999, 2007; Sperandio et al., 1999, 2000; Elliott et al., 2000; Bustamante et al., 2001; Shin et al., 2001; Haack et al., 2003; Porter et al., 2004; Iyoda and Watanabe, 2005; Leverton and Kaper, 2005; Kendall et al., 2011). Regulation of EPEC virulence genes has been demonstrated to involve numerous transcriptional factors and to be influenced by environmental conditions and cell density (Puente et al., 1996; Mellies et al., 1999; Sperandio et al., 1999; Elliott et al., 2000; Shin et al., 2001; Deng et al., 2004; Kaper et al., 2004; Garmendia et al., 2005; Bhatt et al., 2011; Wong et al., 2011).

Investigations of EPEC pathogenesis and virulence factor regulation have primarily used a select few prototype isolates (E2348/69, B171, E22, E110019) (Viljanen et al., 1990; Rasko et al., 2008; Iguchi et al., 2009). Each of the previously used prototype isolates was examined in detail for specific reasons; E2348/69 is an EPEC1 isolate as defined by multi-locus sequence typing (MLST) and has been used in human trials (Levine et al., 1978, 1985; Levine and Rennels, 1978), B171 is an EPEC2 isolate as defined by MLST and has been characterized for plasmid content and infection studies (Riley et al., 1987, 1990; Tobe et al., 1999), E22 is a rabbit adapted EPEC strain that has significant utility in the infection of animal models (Cheney et al., 1980) and E110019 is the prototype isolate of atypical EPEC that had a significantly different clinical presentation in a large Finnish outbreak that appeared to spread within households (Viljanen et al., 1990). The characterization of EPEC virulence-associated genes has also typically involved investigating a limited number of genes at a time, which are usually within virulence gene regions, or are known global regulators in other bacteria (Sperandio et al., 1999, 2000; Bustamante et al., 2001; Haack et al., 2003; Iyoda and Watanabe, 2005; Kendall et al., 2011). In contrast, global transcriptional analysis of bacterial pathogens has provided additional insight into genome-wide transcription during conditions that promote pathogenesis, as well as the identification of novel virulence associated factors (Bergholz et al., 2007; Camarena et al., 2010; Mandlik et al., 2011; Chugani et al., 2012; Sahl and Rasko, 2012). Microarrays have previously been used to identify global gene expression of EHEC in response to nutrient limitation, exposure to host cells, and during multiple growth phases (Bergholz et al., 2007; Jandu et al., 2009; Abu-Ali et al., 2010; Bingle et al., 2014). The regulons of the LEE-encoded regulator (Ler) in EHEC and EPEC prototype isolates was also recently described using microarray analyses (Bingle et al., 2014). Overall, these studies have not provided a global view of the transcription in EPEC.

A new method of investigating global transcriptomes is RNA sequencing (RNA-Seq), which is an unbiased high-throughput sequencing approach used to capture the global transcriptional response of an organism during particular conditions (Mortazavi et al., 2008; Wang et al., 2009; Martin and Wang, 2011; Ozsolak and Milos, 2011). This method allows the simultaneous analysis of all regions of the genome, unlike methods such as microarray or quantitative reverse-transcription PCR (qRT-PCR), which are limited to analyzing known genomic regions as targets. Also, RNA-Seq can be used to analyze isolates that have diverse or unknown genomic content, unlike microarray analysis, which requires that samples exhibit sequence similarity to known targets that were used to develop the probes of the microarray. To date, RNA-Seq has been used to characterize the global transcriptomes of multiple human disease-associated bacteria (Perkins et al., 2009; Camarena et al., 2010; Sharma et al., 2010; Mandlik et al., 2011; Chugani et al., 2012), including enterohemorrhagic *E. coli* (EHEC) (Landstorfer et al., 2014) and enterotoxigenic *E. coli* (ETEC) (Sahl and Rasko, 2012). These studies used RNA-Seq to demonstrate that distinct isolates of *Pseudomonas aeruginosa* have differences in their quorum sensing regulons (Chugani et al., 2012), or to identify genes that are likely acting as global regulators in ETEC (Sahl and Rasko, 2012). However, to our knowledge there have been no prior studies that have used RNA-Seq to describe the global transcriptional response of EPEC. Furthermore, it is not known how much variability exists among the global virulence regulons of EPEC that have emerged in divergent phylogenomic lineages of *E. coli*, each with unique genomic content.

To investigate whether the genomic diversity of four EPEC prototype isolates results in unique transcriptional profiles, an RNA-Seq approach was used to identify the global transcriptomes of these isolates during standard laboratory growth conditions that have been used to study EPEC pathogenesis (Kenny et al., 1997; Sperandio et al., 1999; Leverton and Kaper, 2005). Using a combination of RNA-Seq analysis, comparative transcriptomics and comparative genomics, the current studies demonstrate there are isolate-specific transcriptional responses for different growth conditions including three growth phases and two media types. Differential expression analysis RNA-Seq data from conditions that promote virulence factor expression compared to conditions during which virulence factors are not typically expressed was used to identify all genes that are simultaneously expressed in association with virulence factors. Many of these additional genes encode proteins involved in central metabolism or survival that may indirectly contribute to EPEC pathogenesis. Additional studies in this manuscript examine the bacterial interaction with host cells and identified a transcriptional pattern that is unique to each of the prototype isolates, as well as a core transcriptome under this condition. These studies highlight the diversity in the transcriptional program of these prototype isolates and provide a glimpse into the core transcriptome, which may provide information on genes and gene products that can be targeted as therapeutics and diagnostics for this group of pathogens. The findings of these studies demonstrate the utility of comparative transcriptomics for investigating EPEC. However, a similar approach could be undertaken for any genomically diverse pathogen or species and provide vital information into the transcriptional patterns of pathogens under any number of growth conditions.

## Materials and methods

### Bacterial isolates and media

The bacterial isolates examined in this study were previously characterized and their genome sequences are publicly available (Rasko et al., 2008; Iguchi et al., 2009; Hazen et al., 2013). The genomes were completely sequenced (E2348/69, NC_011601.1- NC_0116013.1) (Iguchi et al., 2009) or sequenced to high quality draft genomes (B171, AAJX00000000; C581-05, AIBE00000000; E110019, AAJW00000000) (Rasko et al., 2008; Hazen et al., 2013). The EPEC isolates were grown in Luria-Bertani (LB) broth medium (Difco) or in Dulbecco's Modified Eagle's Medium (DMEM) supplemented with 4.5 g/L of glucose (Gibco).

### Large scale-BLAST score ratio (LS-BSR)

The four EPEC isolate (E2348/69, B171, C581-05, and E110019) genomes were compared with LS-BSR as previously described (Hazen et al., 2013; Sahl et al., 2014) using TBLASTN (Gertz et al., 2006). The predicted protein-encoding genes of each genome that had ≥90% nucleotide identity to each other were assigned to gene clusters using UCLUST (Edgar, 2010). Representative sequences of each gene cluster were then compared to each genome using TBLASTN (Gertz et al., 2006), and the TBLASTN scores were used to generate a BSR value indicating the detection of each gene cluster in each of the eight genomes. The BSR value was generated by dividing the score of a gene compared to a genome by the score of the gene compared to its own sequence. The representative nucleotide sequence of each gene cluster is provided in Supplemental Data Set 2.

### RNA isolation and sequencing

The EPEC isolates were grown overnight in LB and were inoculated 1:100 into 50 ml of LB, or DMEM supplemented with 4.5 g/L glucose in a 250 ml flask. The flasks were grown at 37°C with shaking (225 rpm) to a final optical density of approximately 0.2, 0.5, or 1.0. Two biological replicates were generated for each EPEC isolate and media type. The cells were concentrated by centrifugation at 3500 rpm for 5 min. Total RNA was isolated using the Ribopure bacteria kit (Ambion) and treated with the Ribopure DNase I to remove contaminating DNA. The samples were then treated with the Turbo DNA-free kit (Ambion) to ensure all contaminating DNA was removed, and the RNA was verified to be DNA free by quantitative PCR (qPCR) analysis using primers for *rpoA*, which encodes the alpha subunit of the RNA polymerase using primers listed in Supplemental Table 1.

RNA-Seq of EPEC cells that have adhered to HeLa was performed as follows. HeLa cells were grown to a 90% confluence in a 6-well culture dish in DMEM supplemented with 4.5 g/L of glucose and 10% fetal bovine serum (FBS). The HeLa cells were washed with fresh DMEM supplemented with 4.5 g/L of glucose lacking FBS, and then 2 ml of the media was added before inoculating overnight cultures of each EPEC isolate 1:100 into the fresh media containing the HeLa cells. The EPEC isolates were allowed to adhere to the HeLa cells for 3 h at 37°C with no shaking. The media containing non-adhered EPEC cells was removed and the HeLa and adhered EPEC cells were rinsed twice with fresh media, then all cells (HeLa and EPEC) were removed by scraping and were resuspended in 1 ml of fresh DMEM. The cells were concentrated via centrifugation at 3500 rpm for 1 min, and total RNA was isolated from both the HeLa and EPEC cells using the MasterPure kit (Epicenter). Contaminating DNA was removed using the Turbo DNA-free kit (Ambion). The polyadenylated HeLa mRNA was depleted from the samples using the GenElute mRNA Miniprep kit (Sigma), which binds poly(A)^+^ labeled mRNA to a spin column. The poly(A)^+^ depleted fraction as well as the eluted poly(A)^+^-enriched fraction were both saved at −80°C for further processing. The poly(A)^+^-depleted fraction was treated again for contaminating DNA using the Turbo DNA-free kit, and was verified to be DNA-free using qPCR as described above. This DNA-free poly(A)^+^-depleted fraction was then submitted for library construction and 100 bp paired-end Illumina sequencing. All of the RNA samples were converted into cDNA libraries using the Ovation Prokaryotic RNA-Seq System (NuGen) that were sequenced on the Illumina HiSeq 2000 to generate 100 bp paired-end reads at the Institute for Genome Sciences Genome Resource Center. The Illumina sequencing reads generated for each RNA sample have been deposited in the short reads archive (SRA), and the accession numbers are listed in Supplemental Table 2.

### RNA-Seq analyses

The Illumina reads generated for each RNA sample were analyzed and compared using an Ergatis-based (Orvis et al., 2010) RNA-Seq analysis pipeline. The completed genome and annotation that is publicly available for EPEC isolate E2348/69 was used for the RNA-Seq analysis of this isolate. The contigs of the draft genome assemblies of B171, C581-05, and E110019 were ordered relative to the completed genome and plasmids of E2348/69 (Iguchi et al., 2009). The contigs of each genome were then combined into several large pseudomolecule sequences, and the protein-encoding regions and other features were predicted using Prodigal (Hyatt et al., 2010) using an in-house annotation pipeline (Galens et al., 2011). The reads for each isolate were aligned to their corresponding genome using Bowtie (Langmead et al., 2009) and the number of reads that aligned to the predicted coding regions were determined using HTSeq (Anders et al., 2014). The differential expression of each gene under two different conditions was determined using DESeq v. 1.5.24 (Anders and Huber, 2010). The reads aligned to each gene were normalized then averaged for each of the two biological replicates. The fold-change and the log_2_ of the fold-change (LFC) were calculated for each of the comparisons. The expression data were then filtered for further analysis and genes that were determined to be transcriptionally altered met the following criteria: LFC ≥2, ≤−2, minimum normalized read count = 10, false discovery rate (FDR) ≤ 0.05 that was determined using DESeq v. 1.5.24 and R-2.15.2. The circular displays of the genes that exhibited significant differential expression were generated using Circos 0.65 (Krzywinski et al., 2009). Heatmaps of the significant LFC values for the LEE and BFP genes were constructed using MeV (Saeed et al., 2006).

The genes in these four isolates are represented by 7493 LS-BSR gene clusters, which were then utilized to examine the expression values for each of these LS-BSR clusters that were conserved among all of the isolates included in this analysis. The analysis was performed using in-house Perl scripts and heatmaps were generated using R statistical package v2.15.2 that in turn utilized the DESeq v1.10.1 library for normalization and the gplots v2.11.0 library for generating the heat maps. The expression values were normalized using the DESeq method (Anders and Huber, 2010). Only 3302 conserved clusters (i.e., represented in all strains) were used to compute the eigenvectors using principal component analysis methods. The first and second principal components were utilized in a scatter plot to visualize the clustering of the strains by gene content and gene expression. The normalized gene expression values were also used to compute the standard deviation for each LS-BSR cluster across all samples and 2000 out of 3302 LS-BSR clusters showing the greatest standard deviations of expression values were utilized to generate a heatmap of the samples using the LS-BSR cluster expressions.

### Quantitative reverse transcriptase PCR (qRT-PCR)

The differences in expression during growth in DMEM compared to LB were determined for genes listed in Supplemental Table 1 using qRT-PCR. RNA that was used for RNA-Seq was reverse transcribed and primed with random hexamers to generate cDNA using the SuperScript III First-Strand Synthesis System for RT-PCR (Invitrogen). The cDNA was diluted 1:20 into nuclease free water (Ambion) before analysis using qPCR. The qPCR on the reverse transcribed RNA samples was performed using SYBR Green master mix (Life Technologies) with 10 μl reactions comprised of the following: 5 μl of 2X SYBR master mix, 1 μl of each of the 5 μM forward and reverse primers (Supplemental Table 1), 1 μl of nuclease free water (Ambion), and 2 μl of cDNA diluted 1:20. Triplicate reactions were performed for each cDNA template and primer combination. The reactions were cycled in a 384-well plate on the 7900HT Fast Real-Time PCR System (Applied Biosystems) using a two-step reaction with an initial incubation of 50°C for 2 min, 95°C for 10 min, then 40 cycles of 95°C for 15 s and 60°C for 1 min, followed by a dissociation stage. The cycle threshold (Ct) values were calculated using the Applied Biosystems software. The Ct values of the biological replicates were averaged and the standard deviation was calculated. The Ct values of target genes of each sample were normalized by subtracting from it the Ct value of the constitutively-expressed RNA polymerase alpha subunit, *rpoA*, resulting in the ΔCt value of a particular gene for each sample. The difference in expression of a target gene (ΔΔCt) in the DMEM compared to the LB samples was then calculated by subtracting the ΔCt of the LB sample from the ΔCt of the DMEM sample. The fold difference of the expression of a particular gene in DMEM compared to LB was determined by calculating 2^−ΔΔ^Ct. The difference in expression is represented in the figures as the log_2_ of the fold-difference (2^−ΔΔ^Ct) for each gene in DMEM compared to LB. The error bars indicate the standard deviation of the ΔΔCt values.

## Results and discussion

### Comparative genomics of the four EPEC prototype isolates

The EPEC prototype isolates selected for global transcriptional analysis belong to four different EPEC phylogenomic lineages as defined in our recent study as well as being from the *E. coli* phylogroups B1 and B2 (Hazen et al., 2013). Three of these isolates (E2348/69, B171, E110019) have been frequently studied as EPEC prototype isolates in research investigating the virulence mechanisms of typical and atypical EPEC (Viljanen et al., 1990; Rasko et al., 2008; Iguchi et al., 2009). The fourth isolate, C581-05, was selected as a representative of the EPEC4 phylogenomic lineage, which is also in phylogroup B2 (Hazen et al., 2013).

**Figure 1:**
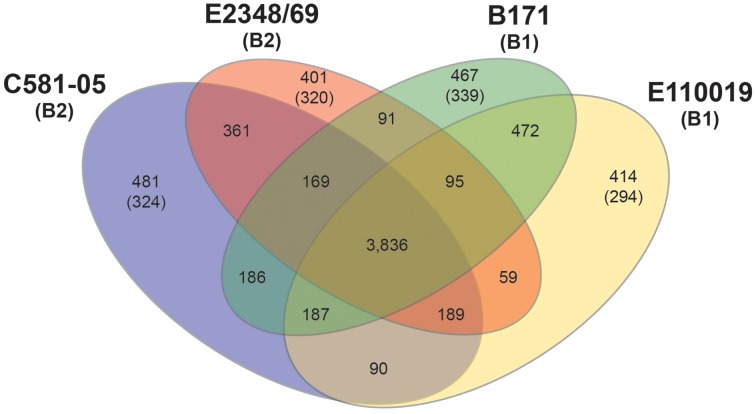
**Comparison of the genomic content of four EPEC prototype isolates**. Venn diagram illustrating the number of shared and unique genes identified among the four EPEC prototype isolates using LS-BSR (Sahl et al., 2014). The *E. coli* phylogroup (Tenaillon et al., 2010; Hazen et al., 2013) of each of the prototype isolates is indicated in parentheses below the isolate name. The number of core genes is indicated, which are genes that are present with significant similarity (LS-BSR ≥ 0.8) in all four of the EPEC isolates. The number of shared genes is indicated in the overlapping regions, which are the genes that have significant similarity (LS-BSR ≥ 0.8) in two or three of the EPEC isolates, but are divergent (LS-BSR < 0.8) or absent (BSR < 0.4) in the other EPEC isolate or isolates. The number of unique genes identified in each EPEC isolate genome are the genes that have LS-BSR ≥ 0.8 in the one genome and <0.8 in the other three genomes. The number of genes in parentheses represents the number of unique genes of each EPEC isolate, which have LS-BSR ≥ 0.8 in the one genome and <0.4 in the other three genomes.

Comparison of the genomic content of the four EPEC prototype isolates (E2348/69, B171, C581-05, E110019) using large-scale BLAST score ratio (LS-BSR) demonstrated there are 3836 genes that are present in all four isolates with significant similarity (LS-BSR ≥ 0.8) (Figure 1). There were a greater number of genes shared between EPEC prototype isolates of the same phylogroup (C581-05 and E2348/69 in phylogroup B2, vs. B171 and E110019 in phylogroup B1) than for isolates of different phylogroups (for example E2348/69 of phylogroup B2, and B171 of phylogroup B1) (Figure 1). In each isolate there was genome content that was not exclusive, but divergent in one or more of the other EPEC isolates. The number of genes that were identified in a single EPEC isolate (LS-BSR ≥ 0.8) that were divergent (LS-BSR < 0.8 but ≥0.4) or absent (LS-BSR < 0.4) in all the other isolates, ranged from 401 to 481 (Figure 1). Each genome also contained isolate-specific content with the number of genes that were unique to a particular isolate and absent from all the other isolates (LS-BSR ≥ 0.8, and <0.4 in all other isolates), ranging from 294 to 339 (Figure 1). These genomic comparisons allowed the determination of the phylogroup- and isolate-specific gene content of the EPEC prototype isolates. These findings were then used to compare the global transcriptomes of these isolates during growth in laboratory conditions that stimulate virulence factor expression.

### RNA-Seq analysis of the global transcriptomes of EPEC prototype isolates

To investigate whether the unique genomic content of each of the EPEC prototype isolates resulted in phylogroup- or isolate-specific differences in the global transcriptomes an RNA-Seq approach was used to analyze each of the prototype isolates grown during standard laboratory conditions. The global transcriptomes were determined for each of the EPEC isolates during growth in rich media (LB), and minimal media (DMEM), which are laboratory conditions that have been previously used to investigate EPEC virulence factor expression. Known virulence factors of EPEC are typically expressed during growth in DMEM but not in LB (Puente et al., 1996; Rosenshine et al., 1996; Leverton and Kaper, 2005). RNA-Seq was also used to investigate the differences in the global transcriptomes during three growth phases (early exponential, late exponential, and stationary phase) corresponding to the optical densities (OD_600_): 0.2, 0.5, and 1.0, respectively (Supplemental Table 2, Table 1, **Figure 3**).

**Table 1:** **Numbers of genes that are differentially-expressed in the EPEC prototype isolates**.

**EPEC isolate**	**BFP content**	**Phylogenomic lineage**	**DE comparisons**	**OD_600_**	**Increased (LFC ≥ 2)^a^**	**Decreased (LFC ≤ −2)^a^**	**Total DE genes^b^**	**No. of DE genes of core clusters^c^**	**No. of DE genes of exclusive clusters^d^**
E2348/69	BFP+	EPEC1	LB vs. LB	0.5 vs. 0.2	312	92	404	347	14
				1.0 vs. 0.5	113	103	216	184	4
				1.0 vs. 0.2	358	184	542	459	18
			DMEM vs. DMEM	0.5 vs. 0.2	26	26	52	42	0
				1.0 vs. 0.5	1	6	7	6	0
				1.0 vs. 0.2	5	3	8	8	0
			DMEM vs. LB	0.2 vs. 0.2	350	438	788	567	49
				0.5 vs. 0.5	203	272	475	356	21
				1.0 vs. 1.0	23	13	36	17	9
			HeLa vs. DMEM	H vs. 0.2	134	247	381	246	56
				H vs. 0.5	76	264	340	204	56
				H vs. 1.0	8	211	219	96	56
B171	BFP+	EPEC2	LB vs. LB	0.5 vs. 0.2	138	49	187	148	2
				1.0 vs. 0.5	75	122	197	146	3
				1.0 vs. 0.2	205	101	306	221	5
			DMEM vs. DMEM	0.5 vs. 0.2	307	115	422	253	15
				1.0 vs. 0.5	72	30	102	76	4
				1.0 vs. 0.2	287	116	403	243	20
			DMEM vs. LB	0.2 vs. 0.2	153	118	271	233	5
				0.5 vs. 0.5	245	276	521	363	18
				1.0 vs. 1.0	379	261	640	418	33
			HeLa vs. DMEM	H vs. 0.2	321	260	581	404	18
				H vs. 0.5	179	262	441	324	18
				H vs. 1.0	268	414	682	460	44
C581-05	BFP+	EPEC4	LB vs. LB	0.5 vs. 0.2	208	65	273	215	10
				1.0 vs. 0.5	207	194	401	300	17
				1.0 vs. 0.2	495	359	854	653	41
			DMEM vs. DMEM	0.5 vs. 0.2	125	33	158	100	12
				1.0 vs. 0.5	728	617	1345	900	116
				1.0 vs. 0.2	885	703	1588	1051	130
			DMEM vs. LB	0.2 vs. 0.2	138	75	213	192	1
				0.5 vs. 0.5	172	265	437	369	5
				1.0 vs. 1.0	473	362	835	520	85
			HeLa vs. DMEM	H vs. 0.2	294	239	533	340	45
				H vs. 0.5	295	333	628	421	50
				H vs. 1.0	754	1055	1809	1196	140
E110019	BFP-	None	LB vs. LB	0.5 vs. 0.2	356	237	593	464	10
				1.0 vs. 0.5	333	214	547	434	10
				1.0 vs. 0.2	639	365	1004	782	35
			DMEM vs. DMEM	0.5 vs. 0.2	108	40	148	118	11
				1.0 vs. 0.5	64	53	117	90	11
				1.0 vs. 0.2	209	97	306	204	32
			DMEM vs. LB	0.2 vs. 0.2	208	130	338	273	25
				0.5 vs. 0.5	223	317	540	427	16
				1.0 vs. 1.0	216	395	611	497	24
			HeLa vs. DMEM	H vs. 0.2	295	333	628	433	65
				H vs. 0.5	310	404	714	555	49
				H vs. 1.0	469	552	1021	749	95

a *LFC is the Log_2_ fold change of the genes that exhibit significant (LFC ≥2 or ≤−2 and FDR ≤ 0.05) differental expression (DE)*.

b *The total number of genes that exhibit significant (LFC ≥ 2 or ≤-2 and FDR ≤ 0.05) DE*.

c *The number of DE genes that belong to core gene clusters (LS-BSR ≥ 0.8 in all four genomes)*.

d *The number of DE genes that belong to gene clusters identified in one genome with an LS-BSR ≥ 0.8 and <0.4 in the other three genomes*.

The total number of Illumina HiSeq reads generated for the 56 RNA samples analyzed was approximately 5.25 billion (Supplemental Table 2). The total number of reads generated for each sample ranged from approximately 73.4 to 153 million, and the percentage of reads that mapped to each corresponding genome sequence ranging from 31 to 83% for the LB and DMEM samples (Supplemental Table 2). The percentage of reads that mapped to each EPEC isolate genome from the HeLa adherence assay samples was decreased, ranging from 28 to 35% (Supplemental Table 2), as would be expected for a sample containing both pathogen and host.

### Principal component analysis reveals isolate- and media-specific patterns of gene expression

**Figure 2:**
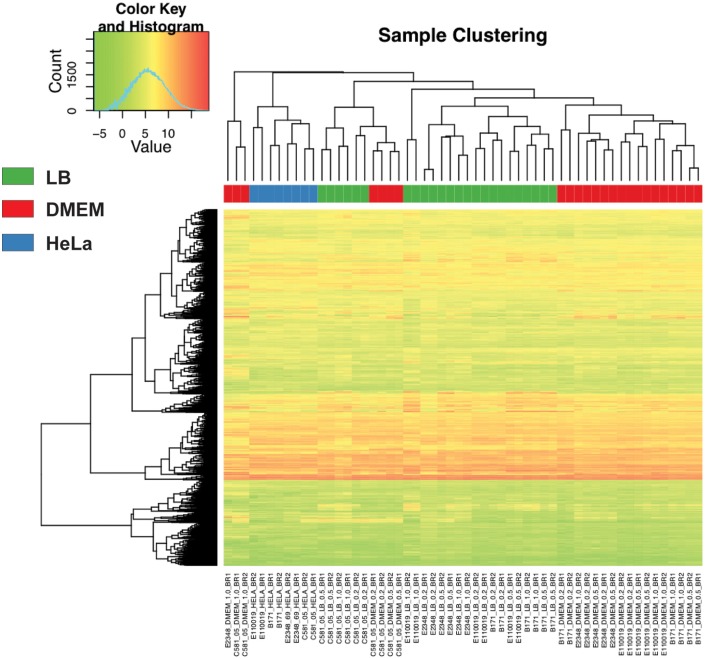
**Comparison of the RNA-Seq samples**. A heatmap with clustering analysis of the expression values was constructed for 2000 LS-BSR gene clusters that were conserved and also expressed in all four EPEC prototype isolates. The normalized gene expression values were used to compute the standard deviation for each LS-BSR gene cluster across all samples. There were 2000 of the 3302 LS-BSR expressed gene clusters that had the greatest standard deviations of expression values that are represented in this heatmap, constructed using the R package gplots v2.11.0. A colored rectangle indicates the media type or experimental treatment.

Principal component analysis of the normalized LS-BSR cluster expression patterns of each RNA-Seq sample demonstrated that there were isolate-specific trends among the samples (Supplemental Figure 1). The samples of B171 and E110019 of phylogroup B1, clustered together while the samples of E2348/69 and C581-05 of phylogroup B2 exhibited less similarity to each other (Supplemental Figure 1). Clustering of samples based on the normalized expression of the conserved LS-BSR clusters that were present in all four of the EPEC isolates also demonstrated similarity among the expression patterns for samples of the same media type with the exception of samples of C581-05 (LB, DMEM, or HeLa) (Figure 2). The samples generated for E2348/69, B171, and E110019 clustered into groups based on growth in the LB or DMEM media, with the exception of the E110019 LB OD_600_ = 1 samples (Figure 2). This suggests that there was a similar transcriptional response in these isolates; however, there were significant differences in the E110019 gene expression during stationary phase in the nutrient rich LB media (Figure 2). The cluster analysis also demonstrated concordance among the biological replicates for all samples with the exception of the replicates of E2348/69 grown in DMEM to early stationary phase (OD_600_ = 1) (Figure 2). We observed that E2348/69 grew slower in DMEM than the other EPEC isolates, which suggests it may have a reduced ability to grow for extended periods of time in the nutrient limiting conditions of DMEM. Interestingly, the C581-05 RNA-Seq samples exhibited divergence from the other EPEC isolate samples, as is demonstrated by the lack of media-specific clustering in the analysis of the expression of conserved genes (Figure 2). The C581-05 LB and DMEM samples grouped together rather than with the samples of the same media type (Figure 2). This suggests that while the EPEC isolates E2348/69, B171, and E110019 exhibited similar overall transcriptional responses to growth in LB and DMEM, C581-05 exhibited a different transcriptional response to growth in the same conditions. The differences in the transcriptional responses of these four EPEC prototype isolates highlights the issues with using a single isolate as the prototype to study the pathogenicity of a pathovar or pathogenic species.

### Isolate-specific changes in gene expression

**Figure 3:**
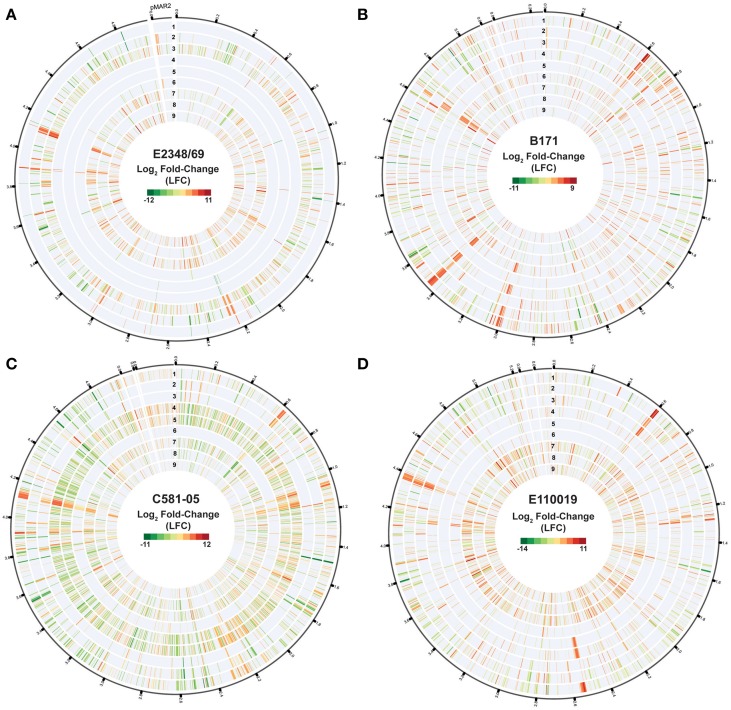
**Isolate- and growth phase-specific differences in the global transcriptomes of the EPEC prototype isolates**. Circular plots of the log_2_ fold-change (LFC) differential expression (DE) values of gene expression when grown to an OD_600_ = 0.5 in DMEM compared to LB, for the EPEC prototype isolates. Each isolate is in a different panel designated as follows: **(A)** E2348/69, **(B)** B171, **(C)** C581-05, and **(D)** E110019. The genes are organized clockwise based on their locus tags for the chromosome and the pMAR2 plasmid of E2348/69, and for the pseudomolecule sequences generated for B171, C581-05, and E110019, which are organized relative to the gene order of E2348/69 as a reference. In each of the circular plots, each of the following tracks numbered sequentially from outside to inside represent the following sample comparisons: DMEM vs. LB OD_600_ = 1.0 (track 1), DMEM vs. LB OD_600_ = 0.5 (track 2), DMEM vs. LB OD_600_ = 0.2 (track 3), DMEM OD_600_ = 1.0 vs. DMEM OD_600_ = 0.2 (track 4), DMEM OD_600_ = 1.0 vs. DMEM OD_600_ = 0.5 (track 5), DMEM OD_600_ = 0.5 vs. DMEM OD_600_ = 0.2 (track 6), LB OD_600_ = 1.0 vs. LB OD_600_ = 0.2 (track 7), LB OD_600_ = 1.0 vs. LB OD_600_ = 0.5 (track 8), LB OD_600_ = 0.5 vs. LB OD_600_ = 0.2 (track 9). The values displayed exhibited significant DE per the following criteria: LFC ≥2, ≤-2, minimum read count = 10, false discovery rate (FDR) ≤ 0.05.

Differential expression analysis of the RNA-Seq samples further demonstrated there were identifiable isolate-specific differences in gene expression during growth in DMEM compared to LB (Table 1, Figure 3 tracks 1–3, Supplemental Data Sets 3–6). The total number of genes that exhibited significant differential expression during growth in DMEM compared to LB ranged from 36 to 835 depending on the growth phase and the EPEC isolate (Table 1). Overall, the EPEC isolates had a greater number of genes that were differentially-expressed in DMEM compared to LB during stationary phase (OD_600_ = 1) than there were during exponential (OD_600_ = 0.5) or early exponential growth (OD_600_ = 0.2) (Table 1, Figure 3 tracks 1–3). However, E2348/69 had the opposite trend with fewer genes that had significant differential expression in DMEM compared to LB during exponential (OD_600_ = 0.5) compared to early exponential growth (OD_600_ = 0.2) (Table 1). These findings demonstrated that *each* of the four EPEC prototype isolates had isolate-specific responses to growth in the nutrient rich LB broth compared to the nutrient limiting conditions of DMEM.

### Growth phase-specific changes in gene expression

The number of differentially-expressed genes also was altered for each of the EPEC isolates when comparing the different growth phases during growth in a single media type (Table 1, Figure 3 tracks 4–9). During growth in DMEM the number of genes that had significant differential expression was greatest for the stationary phase (OD_600_ = 1) samples compared to the early exponential samples (OD_600_ = 0.2), or for the late exponential (OD_600_ = 0.5) compared to the early exponential (OD_600_ = 0.2) samples than observed for the stationary phase (OD_600_ = 1) compared to the late exponential (OD_600_ = 0.5) (Table 1). Overall, E2348/69 had far fewer genes that exhibited significant differential expression when comparing the three different growth phases during growth in DMEM (7–52 genes) than was observed for the other three EPEC isolates during growth in DMEM (range from 102 to 1588 genes) (Table 1, Figure 3 tracks 4–6). The reduced number of genes that exhibited significant differential expression compared to E2348/69 DMEM grown to an OD_600_ = 1 may in part be attributed to the observed decrease in growth of this isolate. The number of differentially-expressed genes when comparing these two growth phases was also reduced when compared to the three other EPEC isolates under the same conditions (Table 1). This indicates that along with a reduced ability to grow for extended periods of time in the DMEM, E2348/69 also had a different transcriptional response to growth in DMEM than was observed for the other EPEC isolates.

### Media-specific changes in gene expression

This data set also allowed the examination of the differences in gene expression in different media. The majority of the genes that had significant differential expression when comparing growth phases were not identified in the comparison of growth in differing media types (Table 2). Overall, there were fewer genes (range 0–501) that exhibited significant altered expression in both LB and DMEM when comparing between the different growth phases (Table 2). This finding highlights the considerable differences in the global transcriptomes of EPEC prototype isolates during growth in nutrient-rich compared to nutrient-limiting media. The greatest number of genes (501) that were differentially-expressed when comparing the different growth phases in both LB and DMEM was identified in the EPEC4 isolate C581-05 (Table 2). In most cases the genes that were differentially-expressed for the different growth phase comparisons of both LB and DMEM had similar trends of increased or decreased expression, and there were fewer genes that were increased during growth in one media type and decreased in the other media type (Table 2). For example, there were only 22 genes of E2348/69 that exhibited significant differential expression in both DMEM and LB when comparing the early exponential (OD_600_ = 0.2) and exponential (OD_600_ = 0.5) growth phases (Table 2). This limited gene set can be partially attributed to the low number of E2348/69 genes (52) that had significant differential expression in DMEM when comparing growth phases (Table 1). Interestingly, nearly all (20 out of 22) of these genes exhibited a different expression trend in LB compared to DMEM, which is in contrast to the other EPEC isolates, which had a greater number of genes with the same trend in expression (Table 2). For each of the E2348/69 genes that had a different trend in expression in the DMEM and LB samples, the expression was increased in the LB samples and decreased in the DMEM samples.

**Table 2:** **Number of genes that are differentially-expressed during different growth phases**.

**EPEC isolate**	**OD_600_**			**No. of differentially-expressed genes^a^**									
		**Total DE genes**		**In LB only**			**In DMEM only**			**In both LB and DMEM**			
		**LB**	**DMEM**	**Total**	**Increased**	**Decreased**	**Total**	**Increased**	**Decreased**	**Total**	**Increased**	**Decreased**	**Different trend**
E2348/69	0.5 vs. 0.2	404	52	382	290	92	30	24	6	22	2	0	20
	1.0 vs. 0.5	216	7	216	113	103	7	1	6	0	0	0	0
	1.0 vs. 0.2	542	8	542	358	184	8	5	3	0	0	0	0
B171	0.5 vs. 0.2	187	422	151	113	38	386	298	88	36	5	7	24
	1.0 vs. 0.5	197	102	185	68	117	90	64	26	12	6	3	3
	1.0 vs. 0.2	306	403	224	137	87	321	239	82	82	40	6	36
C581-05	0.5 vs. 0.2	273	158	221	160	61	106	93	13	52	30	2	20
	1.0 vs. 0.5	401	1345	200	122	78	1144	649	495	201	78	115	8
	1.0 vs. 0.2	854	1588	353	224	129	1087	635	452	501	241	221	39
E110019	0.5 vs. 0.2	593	148	558	330	228	113	81	32	35	25	7	3
	1.0 vs. 0.5	547	117	511	309	202	81	53	28	36	3	4	29
	1.0 vs. 0.2	1004	306	873	535	338	175	108	67	131	96	22	13

a *The number of genes that exhibited significant (LFC ≥ 2 or ≤ −2, FDR ≤ 0.05, ≥ 10 read counts) differential expression at two different growth phases in a single media type (LB or DMEM)*.

For most of the growth phase comparisons, the majority of the differentially-expressed genes were altered only in LB or DMEM and did not exhibit differential expression in both media types (Table 2). The number of genes that were altered only in LB ranged from 151 to 873, while the number of genes that were altered only in DMEM ranged from 7 to 1144 (Table 2). Among the genes that were differentially-expressed when comparing the different growth phases during growth in LB only, were genes involved in metabolism (Supplemental Data Sets 3–6). Meanwhile, genes that were differentially-expressed during different growth phases in DMEM included virulence-associated genes, plasmid-associated genes involved in conjugal transfer, putative phage genes, lipoproteins, and global regulators such as the histone-like protein H-NS, which regulates expression of the LEE (Bustamante et al., 2001; Mellies et al., 2007; Bhatt et al., 2011) (Supplemental Data Sets 3–6). Overall, these studies highlight the transcriptional differences among these four prototype isolates in multiple growth phases and media types.

### Comparison of the global virulence regulons of EPEC prototype isolates during growth in laboratory conditions

**Figure 4:**
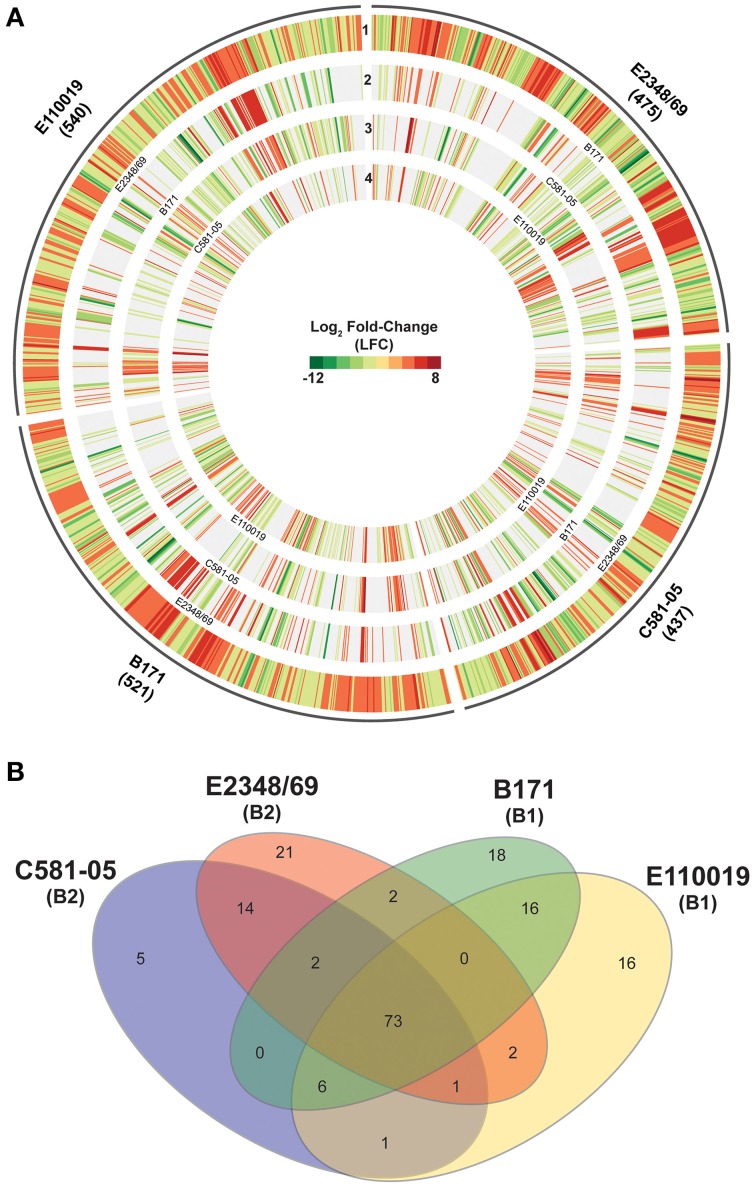
**Comparison of the virulence regulons of the EPEC prototype isolates. (A)** Circular plot comparing the log_2_ fold-change (LFC) values for genes that exhibited significant differential expression (DE) for the EPEC prototype isolates grown to an OD_600_ = 0.5 in DMEM compared to LB. Genes that exhibited significant DE in DMEM compared to LB met the following criteria: LFC ≥2, ≤-2, minimum read count = 10, false discovery rate (FDR) ≤ 0.05. The number of genes that had significant DE is indicated in parentheses below each isolate name. The outermost track of each EPEC isolate displays the LFC values of each of the significant DE genes for that isolate, and the genes are ordered clockwise in the order they appear in the completed or draft genome. Each of the inner tracks displays the LFC values of each gene with significant DE from another EPEC prototype isolate. These significant DE genes have ≥90% nucleotide identity to the corresponding gene in the outermost track. **(B)** Venn diagram showing the number of genes differentially-expressed for each of the four EPEC prototype isolates analyzed in this study grown to an OD_600_ = 0.5 in DMEM compared to LB. The *E. coli* phylogroup (Tenaillon et al., 2010; Hazen et al., 2013) of each of the prototype isolates is indicated in parentheses below the isolate name. The number of core genes is indicated that are highly conserved (LS-BSR ≥ 0.8) in all four of the EPEC isolates that also exhibited significant DE in all four of the EPEC isolates. The number of genes that were identified as highly-conserved (LS-BSR ≥ 0.8) that also exhibited significant DE in two or three EPEC isolates and divergent or absent (LS-BSR < 0.8) is also designated. The number of isolate-specific genes indicates those genes that were exclusive to each EPEC isolate (LS-BSR ≥ 0.4 and <0.4 in all other EPEC isolates) and also exhibited significant DE during growth to an OD_600_ = 0.5 in DMEM compared to LB.

To investigate isolate-specific differences in the expression of known virulence factors, and to identify additional genes that are coordinately-expressed with the known virulence factors, we identified the differences in expression of all protein-encoding genes of the four EPEC prototype isolates during exponential growth in DMEM compared to LB during exponential growth (OD_600_ = 0.5) (Table 1, Supplemental Data Sets 3–6). The total number of genes that exhibited significant differential expression for E2348/69, B171, C581-05, and E110019 were 475, 521, 437, and 540 respectively (Table 1, Figure 4A). These genes include known virulence factors such as those within the LEE and BFP regions (Table 3). However, there were also a number of conserved genes involved in metabolism and stress responses including transporters, and additional isolate-specific genes that represent a potentially unique contribution to the virulence regulon of a particular EPEC isolate (Table 3). A previous study demonstrated there were clade-specific differences in the expression levels of LEE genes encoded by different O157:H7 EHEC isolates during growth in DMEM (Abu-Ali et al., 2010).

**Table 3:** **Select genes that exhibited significant differential expression in DMEM compared to LB at an OD_600_ = 0.5**.

**Gene ID^a^**	**Predicted protein function**	**Protein accession no.^a^**	**Log_2_ fold-change (LFC)^b^^,^^c^**			
			**E2348/69**	**C581-05**	**B171**	**E110019**
**LEE-ENCODED EFFECTORS, REGULATORS, AND ADHESION**						
*eae*	Intimin adherence factor	YP_002331401.1	NS	NS	4.57	4.82
*ler*	Transcriptional regulator	YP_002331430.1	2.81	NS	2.08	NS
*grlR*	Negative regulator GrlR	YP_002331420.1	5.43	NS	3.22	2.33
*grlA*	Positive regulator GrlA	YP_002331419.1	4.89	NS	3.6	NS
*espF*	LEE-encoded effector EspF	YP_002331392.1	5.17	NS	4.7	3.93
*espG*	LEE-encoded effector EspG	YP_002331432.1	4.71	2.67	4.07	3.11
*espH*	LEE-encoded effector EspH	YP_002331406.1	5.64	NS	4.05	2.62
*espZ*	LEE-encoded effector EspZ	YP_002331413.1	4.36	NS	3.31	2.68
*map*	LEE-encoded effector Map	YP_002331404.1	3.87	NS	3.83	2.97
*tir*	Translocated intimin receptor Tir	YP_002331403.1	NS	NS	4.34	3.57
**NON-LEE-ENCODED EFFECTORS**						
*espG*	T3SS secreted effector EspG-like protein	YP_002330404.1	2.56	NS	NS	NS
*nleG*	T3SS secreted effector NleI/NleG-like protein	YP_002328601.1	2.66	NS	NS	NS
*nleH*	T3SS secreted effector NleH-like protein	YP_002328982.1	NS	NS	NS	−2.00
*nleF*	T3SS secreted effector NleF-like protein	YP_002328983.1	NS	NS	NS	−2.40
**EAF PLASMID-ENCODED**						
*bfpA*	Major pilin structural unit bundlin	YP_002332157.1	4.07	NS	3.11	NA
*perA*	Transcriptional activator of the bfp operon	YP_002332173.1	4.67	NS	4.01	NA
*perB*	Transcriptional regulator	YP_002332174.1	4.69	NS	3.69	NA
*perC*	Transcriptional regulator	YP_002332175.1	4.41	−2.04	3.22	NA
**DE GENES OF CORE GENE CLUSTERS**						
*napB*	Citrate reductase cytochrome c-type subunit	YP_002329852.1	−4.11	−3.93	2.14	2.15
*napH*	Quinol dehydrogenase membrane component	YP_002329853.1	−3.45	−3.56	2.48	3.02
*napG*	Quinol dehydrogenase periplasmic component	YP_002329854.1	−3.4	−3.66	2.27	2.69
*rbsD*	D-ribose pyranase	YP_002331517.1	−5.15	2.46	−5.4	−5.27
*cirA*	Colicin I receptor	YP_002329807.1	4.16	5.82	3.62	4.62
*dppF*	Dipeptide transporter ATP-binding subunit	YP_002331254.1	4.55	3.62	2.15	2.84
*espG*	LEE-encoded effector EspG	YP_002331432.1	4.71	2.67	4.07	3.11
*narK*	Nitrate/nitrite transporter	YP_002328887.1	−8.07	−6.81	−2.67	−4.32
*fucI*	L-fucose isomerase	YP_002330550.1	−4.09	−2.45	−3.22	−2.96
*ygeV*	DNA-binding transcriptional regulator	YP_002330601.1	−3.29	−2.42	−4.43	−2.95
*ygfJ*	Hypothetical protein	YP_002330609.1	−5.57	−3.17	−2.32	−2.53
*nanT*	Sialic acid transporter	YP_002330964.1	−3.39	−2.67	−2.12	−2.67
*yjfO*	Biofilm stress and motility protein A	YP_002331964.1	−4.79	−2.21	−4.28	−3.59
**DE GENES OF EXCLUSIVE GENE CLUSTERS**						
**E2348/69**						
*espC*	Serine protease	YP_002330403.1	5.4	NA	NA	NA
*wzx*	O-antigen flippase	YP_002329687.1	5.2	NA	NA	NA
E2348C_2104	Lipoprotein	YP_002329618.1	2.68	NA	NA	NA
**C581-05**						
*idnD*	L-idonate 5-dehydrogenase	WP_024223218.1	NA	−2.02	NA	NA
*tctA*	Tripartite tricarboxylate transporter TctA family protein	YP_543360.1	NA	−2.05	NA	NA
*purR*	Cytochrome C peroxidase	WP_024223889.1	NA	−2.09	NA	NA
**B171**						
EcB171_5053	Glycosyl hydrolase 108 family protein	EDX28171.1	NA	NA	3.56	NA
*bor*	Lipoprotein Bor	EDX30251.1	NA	NA	−2.24	NA
EcB171_3359	Transcriptional regulator, GntR family	EDX31174.1	NA	NA	−2.3	NA
**E110019**						
*imm*	Colicin-E2 immunity protein	EDV85355.1	NA	NA	NA	2.04
EcE110019_3691	Transcriptional regulator, C terminal family protein	EDV86975.1	NA	NA	NA	2.03
*stbB*	Plasmid stability family protein	WP_000361389.1	NA	NA	NA	−2.17

a *The gene symbol or locus id and the protein accession number are indicated for the top match protein. In some cases a protein match could not be identified for a cluster in a particular genome, which likely results from differences in the gene-calling that was used for LS-BSR compared to that used for the GenBank sequences. None indicates there was not a corresponding locus id for the particular genome*.

b *These are LFC values for samples that have been normalized for a single EPEC isolate, and have not been normalized across all EPEC isolates*.

c *NS indicates a value was not significant, while NA indicates a comparison was not applicable*.

To our knowledge there has been no extensive comparison to date of phylogroup- or lineage-specific differences in virulence factor expression among EPEC isolates. Comparison of the differentially-expressed genes of the four prototype isolates that exhibited similarity based on the LS-BSR analysis demonstrated there were isolate-specific differences in the global transcriptomes during exponential growth (OD_600_ = 0.5) in DMEM compared to LB (Figure 4A). This highlights the need to examine multiple isolates and not rely on a single prototype strain for any pathovar to describe an entire group of isolates. Overall, the differentially-expressed genes that were identified in more than one of the EPEC isolates exhibited similar trends of increased or decreased expression (Figure 4A); however, in some instances there were genes identified in multiple EPEC isolates that were increased in some of the isolates and decreased in other isolates (Figure 4A, Table 3). These included some genes identified by LS-BSR as highly-conserved in all four of the EPEC isolates (Supplemental Table 3). Interestingly, several genes (*napB, napG*, and *napH*) of the nap operon, which encodes a periplasmic nitrate reductase (Brondijk et al., 2002), exhibited increased expression in the EPEC isolates of phylogroup B1 (B171 and E110019), but decreased expression in the phylogroup B2 EPEC isolates (E2348/69 and C581-05) (Supplemental Table 3). There were a total of 73 genes that were present in the genomes of all four of the EPEC isolates that were also differentially-expressed in all four EPEC isolates (Figure 4B). Of these 73 genes, there were four that had different trends of expression in these isolates, 18 exhibited consistently increased expression, and 51 exhibited consistently decreased expression in the four EPEC isolates (Supplemental Table 3).

Although there were a number of similarities in the response of the four EPEC prototype isolates to growth in DMEM compared to LB, there were many more genes that exhibited differential expression in one, two or three of the isolates (Figure 4A). The number of genes that were identified by LS-BSR as being unique (LS-BSR ≥ 0.8 and <0.4 in all other isolates) to an EPEC isolate that were differentially-expressed during exponential growth in DMEM compared to LB ranged from only 5–21 genes (Table 1, Figure 4B). The number of genes that were identified in two or three of the EPEC isolates and also had significant differential expression was similarly low, ranging from 0 to 16 genes (Figure 4B). Thus, the majority of the genes that exhibited differential expression in each of the EPEC isolates were genes that were highly-conserved in more than one isolate (Table 1). However, these genes did not exhibit significant differential expression in all four of the isolates (Figure 4B). This may in part be attributed to the stringent cut-offs used in this study for identifying genes with significant differential expression, and with less stringent cut-offs there may be more of these highly-conserved genes with low-level differences identified with significant differential expression in all four isolates.

### Differential-expression of the LEE and BFP regions

**Figure 5:**
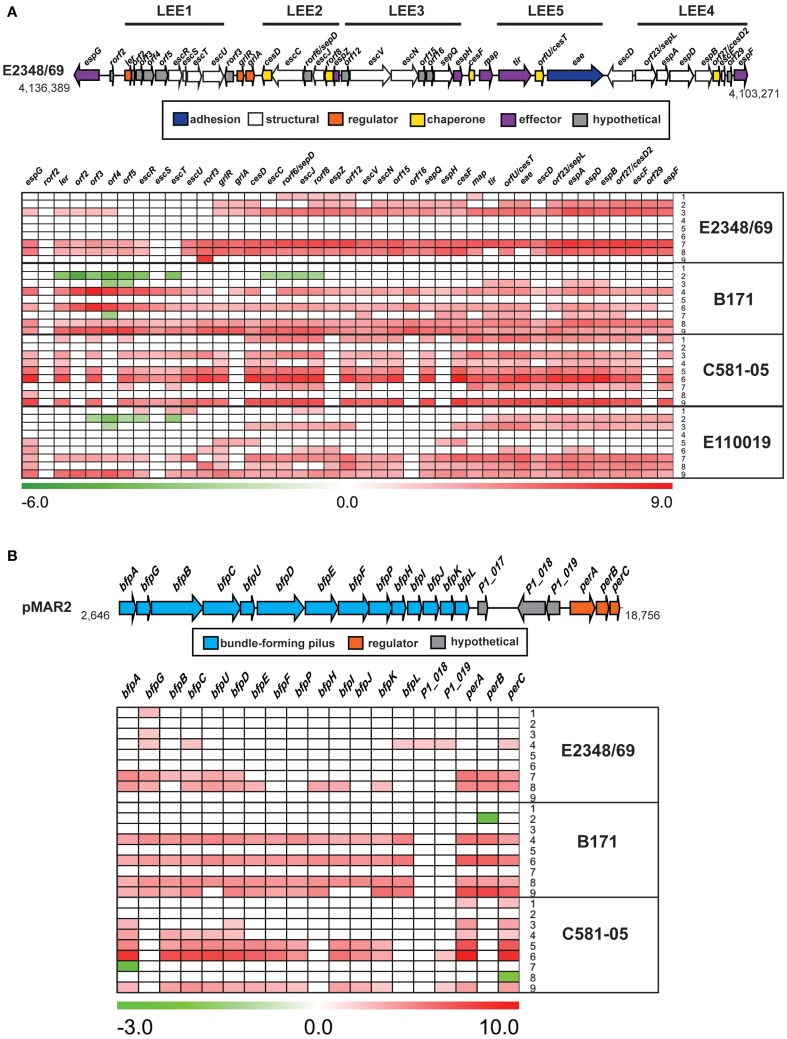
**Comparison of the isolate- and growth phase-specific differences in the expression of genes in the LEE and BFP regions**. A diagram and heatmap of the isolate- and growth phase-specific differences in expression of **(A)** genes encoded within the LEE region of E2348/69, B171, E110019, and C581-05, and **(B)** genes of the BFP operon encoded by the EAF plasmids of E2348/69, B171, and C581-05 (note E110019 naturally lacks the BFP encoding plasmid). Red indicates increased differential expression, green indicates decreased differential expression, and white indicates the difference in expression was not significant or a gene was not present in the EPEC isolate. Each row represents the differential expression (log_2_ fold-change; LFC) for the following different sample comparisons: LB OD_600_ = 0.5 vs. LB OD_600_ = 0.2 (row 1), LB OD_600_ = 1.0 vs. LB OD_600_ = 0.5 (row 2), LB OD_600_ = 1.0 vs. LB OD_600_ = 0.2 (row 3), DMEM OD_600_ = 0.5 vs. DMEM OD_600_ = 0.2 (row 4), DMEM OD_600_ = 1.0 vs. DMEM OD_600_ = 0.5 (row 5), DMEM OD_600_ = 1.0 vs. DMEM OD_600_ = 0.2 (row 6), DMEM vs. LB OD_600_ = 0.2 (row 7), DMEM vs. LB OD_600_ = 0.5 (row 8), and DMEM vs. LB OD_600_ = 1.0 (row 9).

The differential expression of known virulence factors present in all four of the EPEC isolates such as genes encoded by the LEE demonstrated there are isolate-specific differences in the timing and conditions during which the LEE genes are expressed (Figure 5A, Table 3). The majority of the LEE-encoded genes had significantly increased expression during growth in DMEM compared to LB for the exponential growth (OD_600_ = 0.5) and early stationary phase (OD_600_ = 1) samples (Figure 5A). This finding was true for all four of the EPEC isolates except the stationary phase comparison of E2348/69, which was previously noted to have a different gene expression pattern (Figure 2). The increased expression of the intimin gene (*eae*) observed by RNA-Seq analysis was verified using qRT-PCR for these samples (Supplemental Figure 2). Interestingly, the trend of increased LEE gene expression in DMEM compared to LB was not observed for the C581-05 late exponential growth samples, which only exhibited significant expression for one gene, *espG*, in DMEM compared to LB during late exponential growth (OD_600_ = 0.5) (Figure 5A). In contrast, the LEE genes of C581-05 exhibited significant differences in expression during late exponential (OD_600_ = 0.5) compared to early exponential (OD_600_ = 0.2) growth in LB (Figure 5A). This finding suggests that in contrast to other EPEC isolates such as B171, which did not exhibit significant increases in LEE gene expression during growth in LB or DMEM over time, the LEE genes of EPEC isolate C581-05 had increased expression over time during growth in LB (Figure 5A). This potentially suggests an additional role for quorum sensing in the regulation of LEE in C581-05 during growth in nutrient-rich media (LB). This type of variation in gene expression has been previously observed in *E. coli* as being quorum sensing regulated (Sperandio et al., 1999; Sircili et al., 2004) and it is possible that this is the case in these studies; however more detailed analysis of the contribution quorum sensing will require studies that are beyond the scope of the current manuscript. Another notable difference in the expression of LEE genes was identified for both B171 and E110019 of phylogroup B1, which exhibited decreased expression of LEE1 genes during stationary phase growth compared to exponential growth in LB (Figure 5A). This was in contrast to the LEE genes of both E2348/69 and C581-05 of phylogroup B2, which did not exhibit a significant difference in expression of the LEE genes during these conditions (Figure 5A). This further indicates there are phylogroup-specific differences in the transcriptional regulation of EPEC virulence factors.

The genes that were identified with significant genetic conservation and increased gene expression during the virulence-associated conditions in all EPEC isolates, represents the core EPEC virulence regulon. The only LEE-encoded gene that exhibited significant differential expression during exponential growth in DMEM compared to LB in all four of the EPEC isolates was *espG*, which had increased expression in all of the EPEC isolates (Table 3). EspG is a type III secreted effector that alters the cytoskeleton of the host cell by interfering with the function of microtubules and golgi (Hardwidge et al., 2005; Shaw et al., 2005; Tomson et al., 2005; Wong et al., 2011). The increased expression of *espG* in all isolates was verified using qRT-PCR (Supplemental Figure 2). In contrast, few of the non-LEE-encoded T3SS effectors had increased expression during exponential growth in DMEM compared to LB (Table 3), suggesting a lack of global coordination of the regulation of these virulence factors in all isolates.

The differential expression of genes of the BFP region also differed for the three typical EPEC isolates (E2348/69, B171, and C581-05), which encoded the BFP operon (Figure 5B). The majority of the BFP genes exhibited significantly increased expression during exponential growth (OD_600_ = 0.5) in DMEM compared to LB (Figure 5B), which is consistent with the findings of previous studies that EPEC virulence factors exhibit increased expression in DMEM (Puente et al., 1996; Rosenshine et al., 1996; Leverton and Kaper, 2005). Interestingly, EPEC isolate C581-05 did not have significantly increased expression during these conditions, and instead exhibited decreased expression of the transcriptional regulator, *perC* (Figure 5B). PerC has previously been demonstrated to activate transcription of LEE and BFP genes (Gomez-Duarte and Kaper, 1995; Tobe et al., 1996; Bustamante et al., 2011). This finding further suggests that virulence factors such as the BFP genes are constitutively expressed in C581-05 during exponential growth irrespective of media in some isolates. In contrast, the majority of the BFP genes of C581-05 exhibited increased expression during entry into stationary phase in DMEM compared to LB, indicating that BFP expression exhibits a greater increase during the nutrient-limiting conditions over time (Figure 5B). The decreased expression of the plasmid genes (*bfpA* and *perA*) of C581-05 during exponential growth in DMEM compared to LB, and increased expression of these genes during stationary phase was confirmed by qRT-PCR (Supplemental Figure 2). Again, these data suggest a role for quorum sensing in this isolate and highlight the strain specificity of virulence factor regulation in different isolates.

### Differential-expression of EPEC genes not previously identified as virulence factors

Additional genes that exhibited significant differential expression during the virulence-inducing conditions included biotin synthesis genes, dipeptide transporters, and a gene encoding a putative colicin receptor protein (Table 3, Supplemental Table 3). The genes that were determined to have decreased expression in all four EPEC isolates included genes encoding transporters and many genes involved in metabolism (Table 3, Supplemental Table 3). The genes that were identified with significant differential expression in only one of the EPEC isolates included known virulence-associated genes, phage genes, a putative adhesin, lipoproteins, lipopolysaccharide biosynthesis genes, and numerous predicted transcriptional regulators (Table 3, Supplemental Table 3). For example, among the genes that were unique to the E2348/69 transcriptome was *espC*, which encodes a serine protease autotransporter that causes cytotoxicity to host cells and is translocated into the host cell by the type V secretion mechanism (Table 3) (Navarro-Garcia et al., 2004; Vidal and Navarro-Garcia, 2008). There were also several E2348/69 genes involved in O-antigen biosynthesis (*wzy, wzx, wbiO*) that exhibited the greatest increases in expression in DMEM compared to LB (Table 3, Supplemental Table 3). The capsular polysaccharide and O-antigen modification was recently demonstrated to promote pathogenesis of the uropathogenic *E. coli* (UPEC) by increasing their ability to survive in the gut (Sarkar et al., 2014). However, to our knowledge the role of the O-antigen gene expression during EPEC virulence has not been established.

Many of the non-virulence factor genes identified that are co-regulated with the virulence regulons of the EPEC isolates are involved in general metabolism or survival and may enhance the ability of the EPEC isolates to colonize and survive in the human gastrointestinal tract (Table 3). Some of the genes of the *nap* operon (*napB* and *napH)*, which encodes a periplasmic nitrate reductase (Brondijk et al., 2002), were previously determined for the EHEC O157:H7 Sakai strain, to exhibit increased expression during exponential growth in minimal media (Bergholz et al., 2007). Additionally, several metabolic transcriptional regulators have recently been linked to pathogenesis of EHEC and EPEC. For example, a transcriptional regulator, FusR, of O157:H7 EHEC was identified that regulates EHEC metabolism and the expression of LEE and promotes intestinal colonization in response to the presence of the metabolic by-product, fucose, which was identified as being abundant in the mucus layer of the intestine (Pacheco et al., 2012). Also, Cra and KdpE, two other global transcriptional regulators of sugar production and potassium transport, have been demonstrated to also either directly or indirectly regulate expression of LEE genes of EHEC (Njoroge et al., 2012, 2013). Another regulator of glycogen biosynthesis metabolism (Romeo et al., 1993), CsrA, was also previously determined to regulate expression of EPEC virulence factors including the LEE (Bhatt et al., 2009, 2011). Overall, the transcriptional studies demonstrate the interconnectedness of general metabolism and virulence and only through global studies can we begin to understand these relationships.

### Differences in the global transcriptomes of the typical and atypical EPEC prototype isolates

In addition to the phylogroup- and isolate-specific differences in gene expression, there were also differences when comparing the three typical EPEC isolates (E2348/69, B171, and C581-05) to the atypical EPEC isolate (E110019) (Supplemental Data Sets 3–6). The LS-BSR analysis of the EPEC isolate genomic content identified 169 genes that were highly-conserved (LS-BSR ≥ 0.8) among the typical EPEC isolate genomes that were divergent (LS-BSR < 0.8, ≥0.4) or absent (LS-BSR < 0.4) from the atypical EPEC isolate E110019 (Figure 1). Of these 169 genes, there were 20 that were also identified with significant differential expression during growth in DMEM compared to LB in two of the typical EPEC isolates and only two genes that were differentially-expressed in all three of the typical EPEC isolates (Supplemental Data Sets 3–5). The two genes that exhibited significant differential expression in all three of the typical EPEC isolates were *perC* and the lactose permease *lacY* (Supplemental Data Sets 3–5). Meanwhile, genes that were present and exhibited significant differential expression in E110019 that were absent from the typical EPEC isolates included a putative adhesin, a colicin immunity protein, a plasmid stability protein, a phage-associated gene, and numerous conserved hypothetical proteins (Table 3, Supplemental Table 3). These findings suggest that the plasmid maintained in E110019 is also regulated under these conditions, but is not similar to the EAF plasmid in the tEPEC isolates.

### Global transcriptomes of EPEC during adherence to host cells *in vitro*

**Figure 6:**
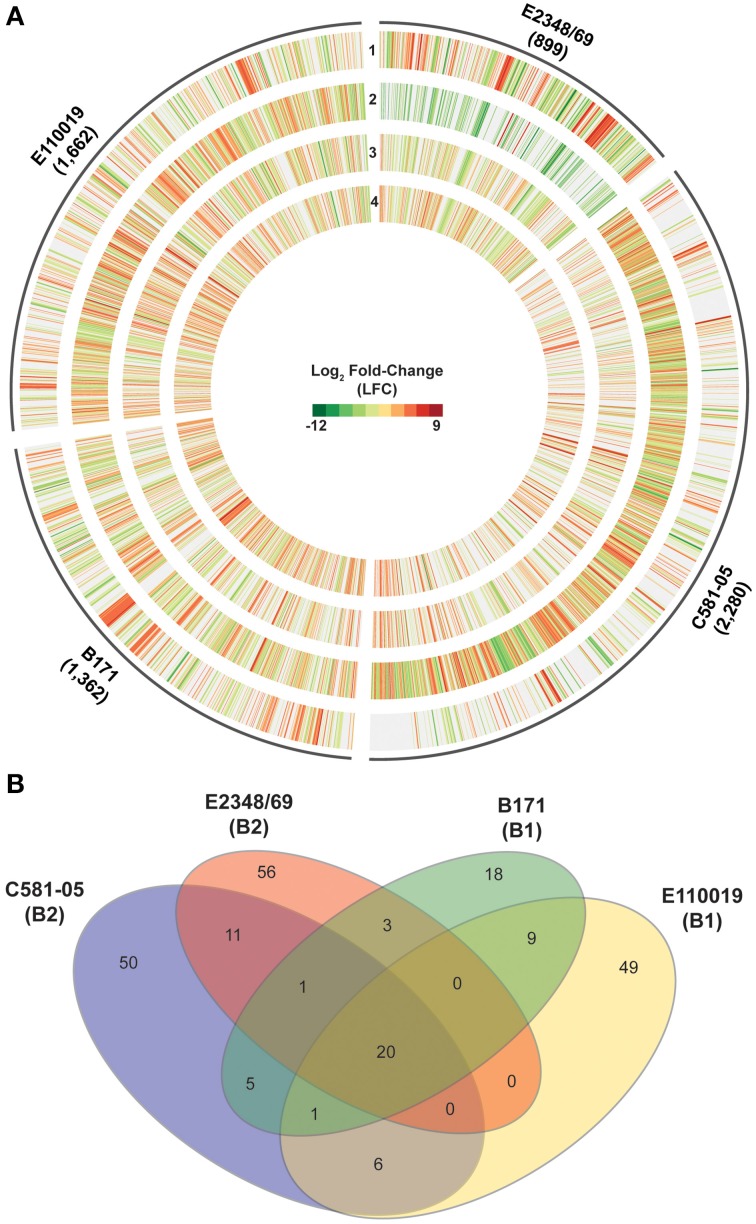
**Comparison of the global transcriptional response of the EPEC prototype isolates during adherence to HeLa cells compared to planktonic growth in DMEM broth. (A)** Circular plot of log_2_ fold-change (LFC) values of genes that exhibited significant differential expression (DE) for each of the EPEC prototype isolates during growth in broth culture or during adherence to HeLa cells during *in vitro* tissue culture assays. The tracks contain LFC values of the following DE comparisons: DMEM OD_600_ = 0.5 vs. LB OD_600_ = 0.5 (track 1), HeLa vs. DMEM OD_600_ = 1.0 (track 2), HeLa vs. DMEM OD_600_ = 0.5 (track 3), and HeLa vs. DMEM OD_600_ = 0.2 (track 4). Red indicates increased DE, green indicates decreased DE, and white indicates the difference in expression was not significant or a gene was not present in the EPEC isolate. **(B)** Venn diagram showing the number of genes differentially-expressed for each of the four EPEC prototype isolates analyzed in this study grown to an OD_600_ = 0.5. The *E. coli* phylogroup (Tenaillon et al., 2010; Hazen et al., 2013) of each of the prototype isolates is indicated in parentheses below the isolate name. The number of core genes is indicated that were highly conserved (LS-BSR ≥ 0.8) in all four of the EPEC isolates and also exhibited significant DE in all four of the EPEC isolates during adherence to HeLa cells compared to growth in DMEM broth culture to an OD_600_ = 0.5. The number of genes that were identified with significant similarity (LS-BSR ≥ 0.8) and also exhibited significant DE in the different combinations of two or three EPEC isolates is also designated. The number of isolate-specific genes indicates those genes that were exclusive to each EPEC isolate (LS-BSR ≥ 0.4 and <0.4 in all other EPEC isolates) and also exhibited significant DE during adherence to HeLa cells compared to growth in DMEM broth culture to an OD_600_ = 0.5.

The mono culture studies described above highlight the variability of the EPEC transcriptome of isolates grown independently of external stimuli. To extend the studies above, the global transcriptome of each EPEC isolate during adherence to HeLa cells compared to growth density were examined (Figure 6A). The total number of genes that exhibited significant differential expression in one or more of these comparisons differed considerably for each EPEC isolate (ranged from 899 to 2280), as indicated by the size of the plot regions and numbers designated in parentheses for each EPEC isolate (Figure 6A, Supplemental Data Sets 3–6).

Comparison of the genes that had significant differential expression during adherence of the EPEC isolates to HeLa cells compared to exponential growth (OD_600_ = 0.5) in DMEM broth demonstrated that most of the altered genes belonged to core gene clusters (Figure 6B). However, there were only 20 genes that were present and also were differentially expressed in all four of the EPEC isolates (Figure 6B, Supplemental Table 4). Furthermore, all but two of these genes had similar trends in terms of increased or decreased expression in all four of the EPEC isolates (Table 4). Among the genes that exhibited increased expression in DMEM compared to LB for all four of the prototype isolates was *hscB*, which has been previously described as a co-chaperone involved in the formation of Fe-S cluster proteins of *E. coli* (Vickery et al., 1997; Hoff et al., 2000). One of the genes that exhibited different trends of expression in the four EPEC isolates was *nirC* (Table 4, Supplemental Table 4), which encodes a protein involved in nitrite uptake in *E. coli* (Clegg et al., 2002). The *nirC* gene was also identified as a virulence-associated factor that increased the survival of *Salmonella* in the presence of macrophages (Das et al., 2009). This finding demonstrates that there are phylogroup-specific responses of EPEC in the presence of host cells, suggesting EPEC isolates of different *E. coli* phylogroups have unique mechanisms for survival during infection, in addition to their differences in virulence factor content that have been previously described (Hazen et al., 2013).

**Table 4:** **Select genes that were differentially-expressed during adherence to HeLa compared to growth in DMEM broth**.

**Gene ID^a^**	**Predicted protein**	**Protein accession no.^a^**	**Log_2_ fold-change (LFC) in HeLa vs. DMEM (OD_600_ = 0.5)^b^^,^^c^**				**LFC in DMEM vs. LB (OD_600_ = 0.5)^b^^,^^c^**
**DE genes of core gene clusters**			**E2348/69**	**C581-05**	**B171**	**E110019**	
*rbsD*	RbsD/FucU transport family protein	YP_002331517.1	−2.21	−4.52	−4.65	−2.96	Decreased in all, except increased in C581-05
*yliE*	Hypothetical protein	YP_002328350.1	−2.42	−6.14	−3.31	−4.27	NS
*slp*	Outer membrane protein Slp	YP_002331206.1	−4.60	−4.62	−3.67	2.73	NS
*yqjB*	SecD export N-terminal TM region family protein	YP_002330857.1	2.28	2.66	2.03	2.13	NS
*hscB*	Fe-S protein assembly co-chaperone HscB	YP_002330308.1	2.17	3.36	3.66	3.28	NS
*nirC*	Putative nitrite transporter	YP_002331085.1	2.60	2.06	−4.90	−7.24	NS
**DE GENES OF EXCLUSIVE GENE CLUSTERS**							
**E2348/69**							
*wzy*	O-antigen polymerase	YP_002329685.1	−5.92	NA	NA	NA	4.39
*rfaS*	Lipopolysaccharide core biosynthesis protein	YP_002331339.1	−6.63	NA	NA	NA	NS
**C581-05**							
*rfaG*	Glycosyl transferases group 1 family protein	WP_024235092.1	NA	−2.14	NA	NA	NS
*argR*	Arginine repressor, DNA binding domain protein	YP_002559154.1	NA	−2.37	NA	NA	NS
*yopT-like*	Cysteine protease, YopT-type domain protein	WP_024235005.1	NA	−2.75	NA	NA	NS
**B171**							
*mhpR*	Mhp operon transcriptional activator	EDX27960.1	NA	NA	2.77	NA	−4.25
*eamA*	EamA-like transporter family protein	WP_011251361.1	NA	NA	2.33	NA	NS
None	Transcriptional regulator, GntR family	EDX31174.1	NA	NA	−2.26	NA	−2.30
**E110019**							
*IutA*	Ferric aerobactin receptor LutA	EDV87696.1	NA	NA	NA	2.56	NS
*terA*	Tellurium resistance family protein	EDV86455.1	NA	NA	NA	−2.42	NS
EcE110019_4492	Sulfatase family protein	EDV86483.1	NA	NA	NA	−3.82	NS

a *The gene symbol or locus id and the protein accession number are indicated for the top match protein. In some cases a protein match could not be identified for a gene cluster in a particular genome, which likely results from differences in the gene-calling that was used for LS-BSR compared to that used for the GenBank sequences. None indicates there was not a corresponding locus id for the particular genome*.

b *These are LFC values for samples that have been normalized for a single EPEC isolate, and have not been normalized across all EPEC isolates*.

c *NS indicates a value was not significant, while NA indicates a comparison was not applicable*.

The number of genes that were exclusive to one of the prototype isolates that also exhibited significant differential expression during adherence to HeLa compared to growth in DMEM media ranged from 18 to 56 genes (Figure 6B). Interestingly, of the 56 genes that were unique to E2348/69 that had significant differential expression, 41 of these genes were previously identified within integrative elements and phages of the E2348/69 chromosome (Iguchi et al., 2009) (Supplemental Table 4). Also, both of the EPEC isolates from phylogroup B2 (E2348/69 and C581-05) were identified with a greater number of genes that were differentially-expressed in HeLa compared to exponential growth (OD_600_ = 0.5) in DMEM broth, than were differentially-expressed in DMEM compared to LB during exponential growth (Table 1). This is in contrast to the EPEC isolates from phylogroup B1 (B171 and E110019), which had similar numbers of genes that were differentially-expressed in the HeLa compared to DMEM, as there were in DMEM compared to LB (Table 1). This finding further indicates that EPEC prototype isolates exhibit phylogroup-specific differences and diverse transcriptional responses to conditions that facilitate pathogenesis.

## Conclusions

In this study, we have demonstrated that RNA-Seq and comparative genomics are powerful tools that can be used together to investigate the global transcriptome of an *E. coli* pathogen that has diverse genomic content. This approach can be used to provide insight into all genes that are simultaneously expressed with virulence factors and may directly or indirectly be involved in EPEC pathogenesis. By investigating differences in the global transcriptomes of EPEC isolates belonging to different evolutionary lineages of *E. coli*, we have demonstrated that genes comprising the unique genomic content of these isolates are in fact differentially-expressed during conditions that promote virulence factor expression. Thus, these studies highlight the need to investigate multiple genomically-diverse isolates rather than a single prototype isolate when investigating virulence mechanisms of a pathovar. Identifying a global transcriptional response that is conserved among divergent EPEC isolates provides insight into the emergence of EPEC isolates in numerous *E. coli* lineages. The conserved transcriptional response of EPEC could be used to develop diagnostic tools to determine whether EPEC are contributing to an infection. This would be particularly useful for patients that carry multiple enteric pathogens, such as was demonstrated in the recent GEMS study (Kotloff et al., 2013) that identified the causative agents of diarrheal illness among young children and infants in numerous study sites in Africa and Asia.

### Conflict of interest statement

The authors declare that the research was conducted in the absence of any commercial or financial relationships that could be construed as a potential conflict of interest.
