# Supplementary material for: RNA-Seq analysis of isolate- and growth phase-specific differences in the global transcriptomes of enteropathogenic Escherichia coli prototype isolates
Source: Front Microbiol. 2015 Jun 12;6:569. doi: 10.3389/fmicb.2015.00569 (PMC4464170; doi:10.3389/fmicb.2015.00569)
Supplement: Supplementary file 1 [file DataSheet1.PDF]

Supplemental Data Files:

Supplemental Data Set 2: Fasta-formatted nucleotide sequences of all gene clusters identified among the four prototype isolates (E2348/69, B171, C581-05, and E110019) using large-scale BLAST score ratio (LS-BSR) analysis.

Supplemental Data Set 3: Differential expression data of *E. coli* isolate E2348/69 for all of the different media and growth phase comparisons.

Supplemental Data Set 4: Differential expression data of *E. coli* isolate B171 for all of the different media and growth phase comparisons.

Supplemental Data Set 5: Differential expression data of *E. coli* isolate C581-05 for all of the different media and growth phase comparisons.

Supplemental Data Set 6: Differential expression data of *E. coli* isolate E110019 for all of the different media and growth phase comparisons.

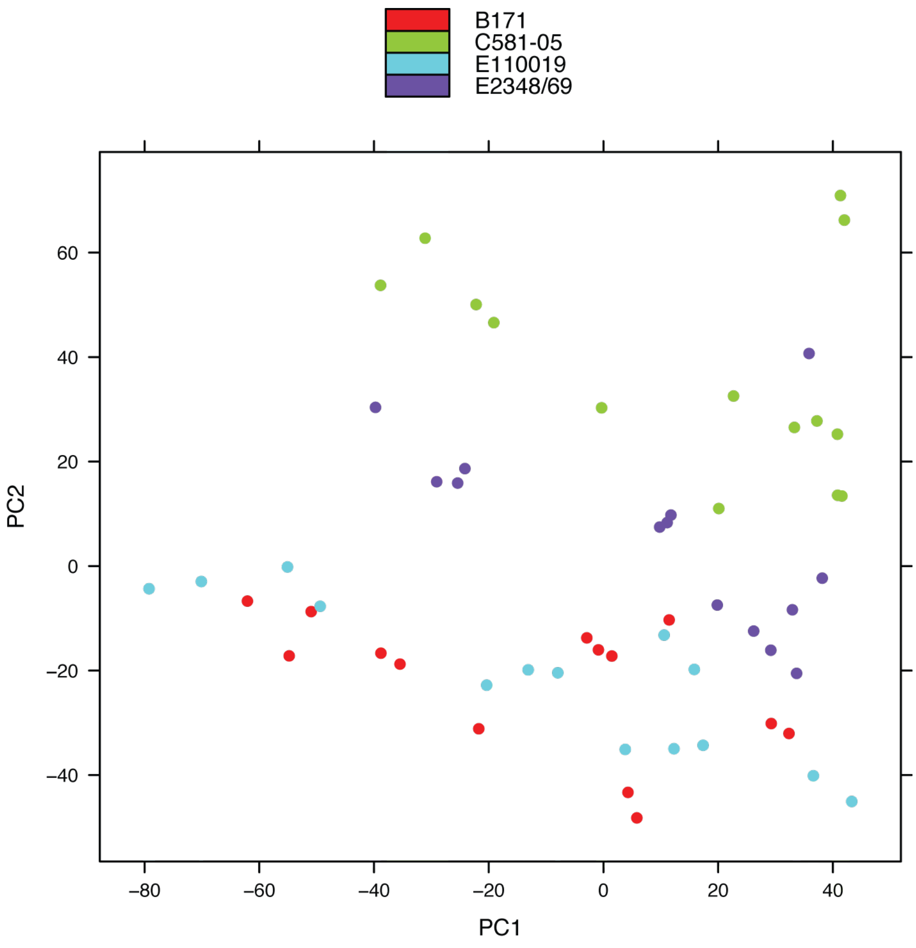

Supplemental Fig. 1

Supplemental Figure 1. Comparison of the RNA-Seq samples. Principal component analysis of the expression of gene clusters identified in all four of the EPEC isolates, represented for all RNA-Seq samples analyzed. There were 3,302 gene clusters identified in all of the EPEC isolates that were used to compute the eigenvectors using principal component analysis. The first and second principal components were utilized in a scatter plot to visualize the clustering of the strains by gene content and gene expression. The samples are colored by EPEC isolate.

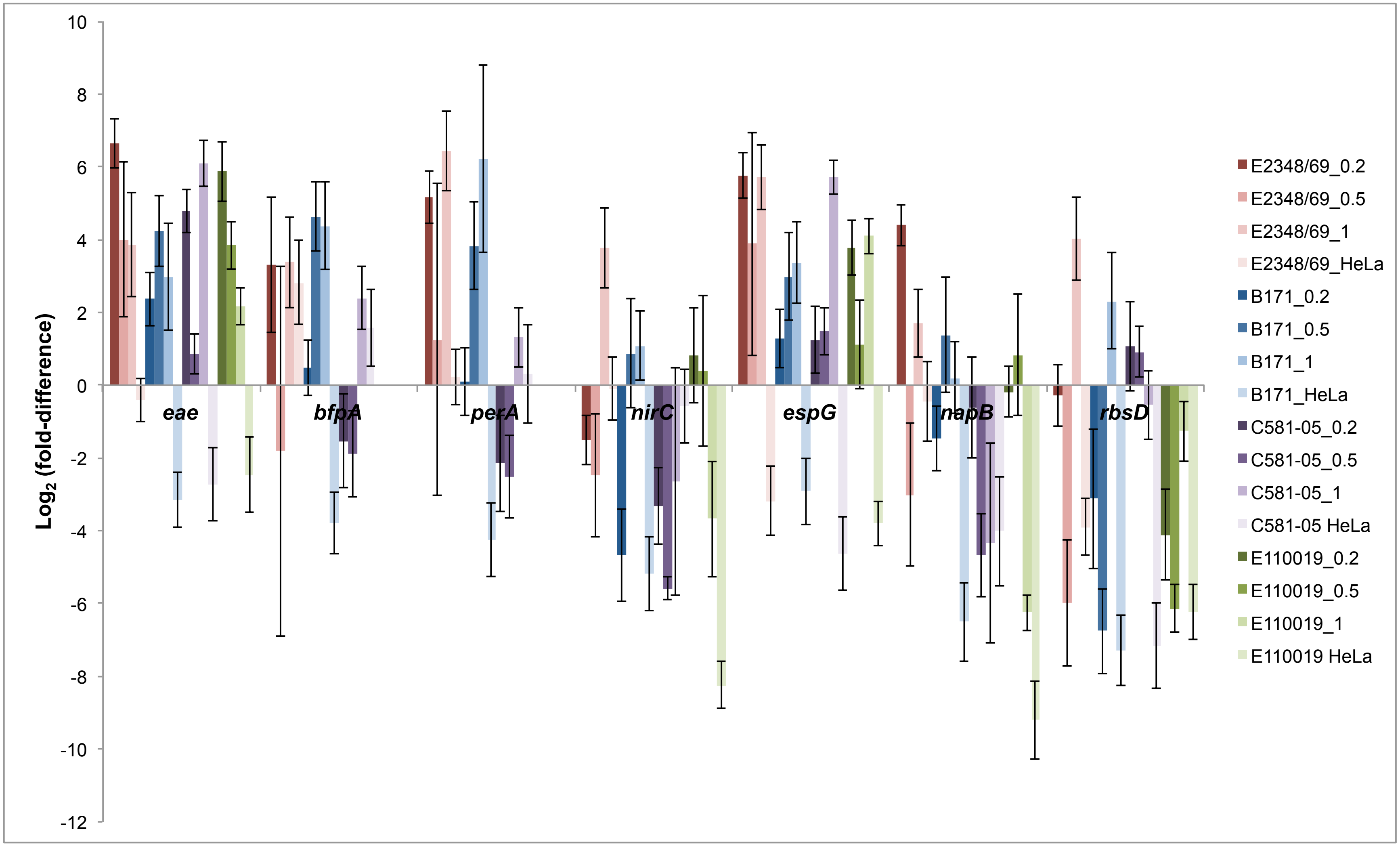

Supplemental Figure 2. Quantitative reverse transcription PCR (qRT-PCR) analysis of select genes for all RNA samples generated in this study. The values represent the $\log_2$ of the fold-difference ($2^{-\Delta\Delta Ct}$) of DMEM samples compared to LB samples for each of the growth phases (OD_{600}= 0.2, 0.5, 1.0), or for cells adhered to HeLa *in vitro* compared to cells grown in DMEM during late exponential growth (OD_{600}= 0.5). The fold-difference

Supplemental Table 1. Primers used in this study

Gene	Predicted Protein Function	Primer ID	Sequence 5'-3'	Source
rpoA	RNA polymerase, alpha subunit	Ec-rpoA-F	TGTAGGCAATACGCTCCACA	(Sahl *et al.* 2012)
		Ec-rpoA-R	GGTTATGTGCCGGCTTCTAC	"
eae	Intimin, attachment protein	eae_361F	TATAGTGCCTTACCTGTCTTAGGTTCGG	This study
		eae_495R	ATAATTTAGAGCCTTGTCATCGGTCGTG	"
bfpA	major subunit of the bundle-forming pilus	bfpA_118F	GCGTCTGATTCCAATAAGTCGCAGAATG	"
		bfpA_380R	GCTTTATCCAACCTGGTAAGCGTCAGAT	"
perA	transcriptional regulator	perA_332F	CTGCGAACCTCAATGAAATGCAATTG	"
		perA_506R	CTGTCTACGATGCTCTTAGATGAAGCAC	"
rbsD	D-ribose pyranase	rbsD_57F	ACATACCGATACGCTGGTGGTGTGT	"
		rbsD_253R	GCAACGTTTCGTGGAGTTGCGGATTA	"
napB	citrate reductase cytochrome c-type subunit	napB_165F	GATGCCGCTGAACTATGTGAATCAGCC	"
		napB_328R	CGTCGCTGTCCATAAAGTGAGTAGGACT	"
nirC	putative nitrite transporter	nirC_190F	ACCTTTGGTATCGCCTTAACGCTGG	"
		nirC_357R	AACGAAGACGGAACCGACCAGGTTAC	"
espG	Type III secreted effector	espG_299F	CAGATGGCTCATCCTTAAGAATTTCCGTCAC	"
		espG_468R	ACGATGAACCTGAATGTATGGCATGTGG	"

Sahl, J.W., and Rasko, D.A. (2012). Analysis of Global Transcriptional Profiles of Enterotoxigenic Escherichia coli Isolate E24377A. Infect Immun. 80, 1232-1242.

Supplemental Table 2. Characteristics of the RNA-Seq samples generated in this study

Strain Id	Condition[a]	OD600	Replicate	Total Reads	Total Reads Mapped	Percent Mapped
E2348/69	LB	0.21	1	103,912,348	67,971,320	65.41
		0.23	2	137,436,474	88,578,440	64.45
		0.50	1	84,974,348	58,955,382	69.38
		0.51	2	103,395,094	70,030,950	67.73
		0.99	1	92,848,676	64,702,984	69.69
		0.98	2	132,577,644	92,965,946	70.12
	DMEM	0.24	1	88,098,420	61,336,642	69.62
		0.21	2	94,374,690	66,608,854	70.58
		0.52	1	97,821,086	73,595,802	75.24
		0.52	2	91,319,090	76,283,672	83.54
		1.04	1	86,014,144	50,183,286	58.34
		1.04	2	119,409,804	69,990,290	58.61
	HeLa	NA	1	54,637,046	17,883,822	32.73
		NA	2	52,642,424	18,922,780	35.95
B171	LB	0.23	1	103,912,348	34,854,618	33.54
		0.23	2	99,578,990	34,436,750	34.58
		0.50	1	97,886,854	58,605,846	59.87
		0.53	2	95,162,186	53,323,662	56.03
		1.01	1	90,207,950	36,265,700	40.20
		1.03	2	85,546,094	32,926,456	38.49
	DMEM	0.23	1	106,737,048	36,427,298	34.13
		0.22	2	112,448,180	35,163,190	31.27
		0.5	1	96,414,722	53,218,754	55.20
		0.52	2	83,297,048	36,132,262	43.38
		1.01	1	106,770,366	32,762,434	30.68
		1.06	2	105,914,360	36,450,488	34.42
	HeLa	NA	1	51,751,624	19,997,404	38.64
		NA	2	57,722,148	21,685,112	37.57
C581-05	LB	0.20	1	138,262,828	65,729,310	47.54
		0.23	2	105,310,116	50,775,228	48.21
		0.50	1	79,631,062	37,601,852	47.22
		0.55	2	153,095,968	69,746,256	45.56
		1.02	1	85,062,468	51,475,984	60.52
		0.99	2	73,393,418	43,352,644	59.07
	DMEM	0.20	1	86,098,528	47,300,918	54.94
		0.21	2	95,395,172	53,895,228	56.50
		0.51	1	89,573,524	52,417,862	58.52
		0.50	2	96,669,160	56,712,030	58.67
		1.03	1	76,827,966	42,545,518	55.38
		1.05	2	87,205,256	50,047,514	57.39
	HeLa	NA	1	49,183,448	15,772,610	32.07
		NA	2	52,485,072	16,586,484	31.60
E110019	LB	0.20	1	118,777,820	72,592,788	61.12
		0.20	2	93,985,728	51,046,152	54.31
		0.54	1	89,344,990	52,135,318	58.35
		0.54	2	121,376,984	70,701,032	58.25
		1.02	1	85,062,468	41,826,560	49.17
		0.99	2	104,946,892	51,813,238	49.37
	DMEM	0.25	1	106,943,552	61,822,458	57.81
		0.24	2	108,831,334	58,461,256	53.72
		0.51	1	73,908,286	23,548,930	31.86
		0.53	2	115,823,816	45,207,916	39.03
		1.04	1	114,224,294	43,695,758	38.25
		0.99	2	106,648,760	36,892,170	34.59
	HeLa	NA	1	52,803,682	17,793,276	33.70
		NA	2	59,459,004	16,760,456	28.19

[a]The experimental conditions for cells grown in either Luria-Bertani (LB) broth, Dulbecco's Modified Eagle's Medium (DMEM) with high glucose grown to an OD_{600} of 0.2, 0.5, or 1.0, or during adherence to HeLa during *in vitro* tissue culture.

Supplemental Table 3. Genes that are shared or unique to the four EPEC prototype strains and have significant differential expression during growth in DMEM

LS-BSR Cluster ID	Gene ID[a]	Predicted Protein	Protein Accession No.[a]
DE Genes of the Core Clusters			
Cluster_1587	*treB*	PTS system trehalose(maltose)-specific transporter subunit IIBC	YP_002332016.1
Cluster_4243	*treC*	trehalose-6-phosphate hydrolase	YP_002332015.1
Cluster_4090	*yjfO*	biofilm stress and motility protein A	YP_002331964.1
Cluster_4250	*eptA*	cell division protein	YP_002331895.1
Cluster_2996	*lamB*	maltoporin	YP_002331805.1
Cluster_850	*glpF*	glycerol facilitator	YP_002331689.1
Cluster_1282	*glpK*	glycerol kinase	YP_002331688.1
Cluster_2452	*fdoH*	formate dehydrogenase-O, Fe-S subunit FdoH	YP_002331654.1
Cluster_161	*fdoI*	formate dehydrogenase-O subunit gamma	YP_002331653.1
Cluster_2927	*tnaA*	tryptophanase	YP_002331479.1
Cluster_4938	*lldD*	L-lactate dehydrogenase	YP_002331316.1
Cluster_6242	*lldR*	DNA-binding transcriptional repressor LldR	YP_002331315.1
Cluster_4241	*lldP*	L-lactate permease	YP_002331314.1
Cluster_5913	*yhiP*	inner membrane transporter YhiP	YP_002331199.1
Cluster_3300	E2348C_3671	hypothetical protein	YP_002331138.1
Cluster_5887	*glpD*	glycerol-3-phosphate dehydrogenase	YP_002331137.1
Cluster_3054	*nanA*	N-acetylneuraminate lyase	YP_002330965.1
Cluster_1357	*nanT*	sialic acid transporter	YP_002330964.1
Cluster_2948	*nanE*	N-acetylmannosamine-6-phosphate 2-epimerase	YP_002330963.1
Cluster_1492	*agaV*	PTS system N-acetylgalactosamine-specific transporter subunit IIB	YP_002330885.1
Cluster_173	*garL*	alpha-dehydro-beta-deoxy-D-glucarate aldolase	YP_002330880.1
Cluster_2501	*tdcA*	DNA-binding transcriptional activator TdcA	YP_002330876.1
Cluster_4979	*tdcB*	threonine dehydratase	YP_002330875.1
Cluster_4351	*tdcC*	threonine/serine transporter TdcC	YP_002330874.1
Cluster_5935	*tdcD*	propionate/acetate kinase	YP_002330873.1
Cluster_5990	*tdcE*	pyruvate formate-lyase 4/2-ketobutyrate formate-lyase	YP_002330872.1
Cluster_2546	*yqhD*	alcohol dehydrogenase	YP_002330766.1
Cluster_7187	*nupG*	nucleoside transporter	YP_002330693.1
Cluster_5123	*ygfJ*	hypothetical protein	YP_002330609.1
Cluster_6138	*ygeV*	DNA-binding transcriptional regulator	YP_002330601.1
Cluster_2019	*fucI*	L-fucose isomerase	YP_002330550.1
Cluster_994	*glpC*	sn-glycerol-3-phosphate dehydrogenase subunit C	YP_002329890.1
Cluster_2741	*glpB*	anaerobic glycerol-3-phosphate dehydrogenase subunit B	YP_002329889.1
Cluster_2273	*glpA*	sn-glycerol-3-phosphate dehydrogenase subunit A	YP_002329888.1
Cluster_824	*glpT*	sn-glycerol-3-phosphate transporter	YP_002329887.1
Cluster_691	*glpQ*	glycerophosphodiester phosphodiesterase	YP_002329886.1
Cluster_2299	*mglB*	methyl-galactoside transporter subunit	YP_002329802.1
Cluster_1511	*mglA*	galactose/methyl galaxtoside transporter ATP-binding protein	YP_002329801.1
Cluster_4984	*mglC*	beta-methylgalactoside transporter inner membrane protein	YP_002329800.1
Cluster_2562	*yeiA*	dihydropyrimidine dehydrogenase	YP_002329799.1
Cluster_2554	*yeiT*	oxidoreductase	YP_002329798.1
Cluster_6152	*ydeQ*	fimbrial protein-like protein	YP_002329152.1
Cluster_4202	*ydeN*	hypothetical protein	YP_002329148.1
Cluster_4386	*narG*	nitrate reductase 1 subunit alpha	YP_002328888.1
Cluster_4345	*narK*	nitrate/nitrite transporter	YP_002328887.1
Cluster_5514	*putA*	trifunctional transcriptional regulator/proline dehydrogenase/pyrroline-5-carboxylate dehydrogenase	YP_002328625.1
Cluster_5925	*agp*	glucose-1-phosphatase/inositol phosphatase	YP_002328612.1
Cluster_3615	*ykgG*	transporter	YP_002327851.1
Cluster_7109	*ykgF*	amino acid dehydrogenase with NAD(P)-binding domain and ferridoxin-like domain	YP_002327850.1
Cluster_6578	*ykgE*	oxidoreductase	YP_002327849.1
Cluster_909	*yicG*	hypothetical protein	YP_002331372.1
Cluster_2166	*rbsD*	D-ribose pyranase	YP_002331517.1
Cluster_2388	*purD*	phosphoribosylamine--glycine ligase	YP_002331768.1
Cluster_4856	*espG*	LEE-encoded effector EspG	YP_002331432.1
Cluster_4265	*dppA*	dipeptide transporter	YP_002331258.1
Cluster_4947	*dppB*	dipeptide transporter permease DppB	YP_002331257.1
Cluster_7336	*dppC*	dipeptide transporter	YP_002331256.1
Cluster_4718	*dppD*	dipeptide transporter ATP-binding subunit	YP_002331255.1
Cluster_6361	*dppF*	dipeptide transporter ATP-binding subunit	YP_002331254.1
Cluster_4390	*gltB*	glutamate synthase subunit alpha	YP_002330959.1
Cluster_2920	*purN*	phosphoribosylglycinamide formyltransferase	YP_002330223.1
Cluster_6411	*purM*	phosphoribosylaminoimidazole synthetase	YP_002330222.1
Cluster_2628	*mntH*	manganese transport protein MntH	YP_002330085.1
Cluster_6868	*cirA*	colicin I receptor	YP_002329807.1
Cluster_4924	*purT*	phosphoribosylglycinamide formyltransferase 2	YP_002329493.1
Cluster_3566	*bioD*	dithiobiotin synthetase	YP_002328297.1
Cluster_2844	*bioC*	biotin biosynthesis protein BioC	YP_002328296.1
Cluster_4872	*bioF*	8-amino-7-oxononanoate synthase	YP_002328295.1
Cluster_6387	*bioB*	biotin synthase	YP_002328294.1
Cluster_3972	*ybdB*	hypothetical protein	YP_002328071.1
Cluster_350	*napG*	quinol dehydrogenase periplasmic component	YP_002329854.1
Cluster_165	*napH*	quinol dehydrogenase membrane component	YP_002329853.1
Cluster_1067	*napB*	citrate reductase cytochrome c-type subunit	YP_002329852.1
DE genes of exclusive gene clusters			
E2348/69			
Cluster_5510	*espC*	serine protease	YP_002330403.1
Cluster_5926	*wzx*	O-antigen flippase	YP_002329687.1
Cluster_2891	*wbiO*	O-acetyltransferase	YP_002329686.1
Cluster_5605	E2348C_2919	hypothetical protein	YP_002330407.1
Cluster_5755	E2348C_2094	hypothetical protein	YP_002329608.1
Cluster_7154	*wzy*	O-antigen polymerase	YP_002329685.1
Cluster_5439	E2348C_2920	hypothetical protein	YP_002330408.1
Cluster_2060	E2348C_2493	hypothetical protein	YP_002329997.1
Cluster_4687	*wbiQ*	fucosyltransferase	YP_002329683.1
Cluster_3482	*wbiP*	glycosyl transferase family protein	YP_002329684.1

Cluster_1339	E2348C_2918	hypothetical protein	YP_002330406.1
Cluster_7180	E2348C_2705	glycosyl transferase family protein	YP_002330203.1
Cluster_3393	E2348C_2104	lipoprotein	YP_002329618.1
Cluster_4066	E2348C_3787	transcriptional regulator, XRE family	YP_002331253.1
Cluster_3662	E2348C_0669	hypothetical protein	YP_002328238.1
Cluster_2499	E2348C_2105	hypothetical protein	YP_002329619.1
Cluster_1612	E2348C_2917	hypothetical protein	YP_002330405.1
Cluster_3679	E2348C_0722	hypothetical protein	YP_002328289.1
Cluster_2002	E2348C_3896	hypothetical protein	YP_002331358.1
Cluster_4291	*atlT*	D-arabitol membrane transporter	YP_002329739.1
Cluster_2827	*atlD*	D-arabitol dehydrogenase	YP_002329741.1
C581-05			
Cluster_5617	None	conserved hypothetical protein	NP_754375.1
Cluster_7481	*idnD*	L-idonate 5-dehydrogenase	WP_024223218.1
Cluster_4198	*tctA*	tripartite tricarboxylate transporter TctA family protein	YP_543360.1
Cluster_6285	*purR*	cytochrome C peroxidase	WP_024223889.1
Cluster_4524	None	hypothetical protein	EMV21983.1
B171			
Cluster_3984	EcB171_5069	putative protein gp35	EDX28188.1
Cluster_3397	EcB171_5053	glycosyl hydrolase 108 family protein	EDX28171.1
Cluster_7340	EcB171_5068	mu-like prophage major head subunit gpT family protein	EDX28180.1
Cluster_6308	EcB171_5067	mu-like prophage I family protein	EDX28179.1
Cluster_4046	EcB171_5070	conserved hypothetical protein	EDX28190.1
Cluster_3404	EcB171_5071	conserved hypothetical protein	EDX28192.1
Cluster_3909	None	hypothetical protein	ELF15994.1
Cluster_6793	None	conserved hypothetical protein	WP_000065111.1
Cluster_7065	EcB171_4952	conserved hypothetical protein	EDX27900.1
Cluster_1800	*bor*	lipoprotein Bor	EDX30251.1
Cluster_3452	EcB171_3359	transcriptional regulator, GntR family	EDX31174.1
Cluster_5599	EcB171_5713	hypothetical protein	EDX27745.1
Cluster_3366	EcB171_3360	phosphoenolpyruvate-dependent sugar phosphotransferase system, EIIA 2 family protein	EDX31157.1
Cluster_1839	EcB171_3361	PTS system, Lactose/Cellobiose specific IIB subunit	EDX31248.1
Cluster_7388	EcB171_2806	polysaccharide deacetylase family protein	EDX31671.1
Cluster_7407	EcB171_2807	glycosyl transferases group 1 family protein	EDX31599.1
Cluster_7466	EcB171_2808	conserved hypothetical protein	EDX31707.1
Cluster_4761	*mhpR*	bacterial transcriptional regulator family protein	EDX27960.1
E110019			
Cluster_4631	None	bifunctional enterobactin receptor/adhesin protein	WP_001223350.1
Cluster_1750	EcE110019_2425	conserved hypothetical protein	EDV85382.1
Cluster_3743	None	conserved hypothetical protein	WP_021548058.1
Cluster_6780	EcE110019_1621	hypothetical protein	EDV87682.1
Cluster_5454	EcE110019_3690	conserved hypothetical protein	EDV86997.1
Cluster_3271	None	phage major capsid protein, HK97 family	WP_001137344.1
Cluster_2033	*imm*	colicin-E2 immunity protein	EDV85355.1
Cluster_6248	EcE110019_3691	transcriptional regulator, C terminal family protein	EDV86975.1
Cluster_6635	None	conserved hypothetical protein	WP_000516614.1
Cluster_7018	*yajA*	putative YajA protein	EDV85356.1
Cluster_4797	EcE110019_4792	putative acetyltransferase	EDV90085.1
Cluster_6980	*stbB*	plasmid stability family protein	WP_000361389.1
Cluster_7104	EcE110019_1001	RNA-directed DNA polymerase	EDV86222.1
Cluster_3692	None	conserved hypothetical protein	WP_021569216.1
Cluster_7206	EcE110019_4502	conserved hypothetical protein	EDV86430.1
Cluster_6289	EcE110019_4438	glycosyl transferase	EDV86461.1

[a]The gene symbol or locus id and the protein accession number are indicated for the top match protein. In some cases a protein match could not be identifed for likely results from differences in the gene-calling that was used for LS-BSR compared to that used for the GenBank sequences. None indicates there was not a c genome.

Supplemental Table 4. Genes that are shared or unique to the four EPEC prototype strains and have significant differential expression during adherence to HeLa compared to growth in DMEM to an OD_{600} of 0.5

LS-BSR Cluster ID	Gene ID[a]	Predicted Protein	Protein Accession No.[a]	DE Trend
DE genes of core gene clusters				
Cluster_4794	*nirC*	nitrite transporter NirC	YP_002331085.1	increased (B2), decreased (B1)
Cluster_2242	*aidB*	isovaleryl CoA dehydrogenase	YP_002331962.1	increased
Cluster_7429	*cdaR*	carbohydrate diacid transcriptional activator CdaR	YP_002327756.1	increased
Cluster_563	*hscB*	co-chaperone HscB	YP_002330308.1	increased
Cluster_6300	*proW*	glycine betaine transporter membrane protein	YP_002330430.1	increased
Cluster_6863	*ptrB*	protease 2	YP_002329489.1	increased
Cluster_252	*yebE*	hypothetical protein	YP_002329490.1	increased
Cluster_1131	*yjfN*	hypothetical protein	YP_002331963.1	increased
Cluster_1142	*yqjB*	hypothetical protein	YP_002330857.1	increased
Cluster_5259	*asnA*	asparagine synthetase AsnA	YP_002331513.1	decreased
Cluster_4996	*dhaK*	dihydroxyacetone kinase subunit DhaK	YP_002328864.1	decreased
Cluster_6631	*dhaL*	dihydroxyacetone kinase subunit DhaL	YP_002328863.1	decreased
Cluster_1222	*dhaM*	dihydroxyacetone kinase subunit DhaM	YP_002328862.1	decreased
Cluster_6893	*malP*	maltodextrin phosphorylase	YP_002331129.1	decreased
Cluster_1158	*ptsI*	phosphoenolpyruvate-protein phosphotransferase	YP_002330102.1	decreased
Cluster_2166	*rbsD*	D-ribose pyranase	YP_002331517.1	decreased
Cluster_5053	*slp*	outer membrane lipoprotein	YP_002331206.1	decreased, except increased in E110019
Cluster_4888	*ycdO*	hypothetical protein	YP_002328628.1	decreased
Cluster_7467	*yjiA*	GTP-binding protein YjiA	YP_002332094.1	decreased
Cluster_4619	*yliE*	hypothetical protein	YP_002328350.1	decreased
DE genes of exclusive gene clusters				
E2348/69				
Cluster_1954	E2348C_2647	hypothetical protein	YP_002330146.1	5.03
Cluster_2743	E2348C_1358	hypothetical protein	YP_002328898.1	2.92
Cluster_7298	E2348C_2632	DNA transposition protein	YP_002330131.1	2.69
Cluster_3278	E2348C_2227	phosphatase	YP_002329738.1	2.02
Cluster_6962	E2348C_0721	hypothetical protein	YP_002328288.1	-2.04
Cluster_6944	E2348C_3648	hypothetical protein	YP_002331115.1	-2.13
Cluster_2750	E2348C_1635	hypothetical protein	YP_002329158.1	-2.14
Cluster_7209	E2348C_1636	hypothetical protein	YP_002329159.1	-2.30
Cluster_1339	E2348C_2918	hypothetical protein	YP_002330406.1	-2.30
Cluster_7384	E2348C_0671	hypothetical protein	YP_002328240.1	-2.31
Cluster_6253	E2348C_0995	DNA-damage-inducible protein	YP_002328558.1	-2.37
Cluster_7470	E2348C_0815	hypothetical protein	YP_002328381.1	-2.46
Cluster_7494	E2348C_2737	hypothetical protein	YP_002330235.1	-2.60
Cluster_5285	E2348C_2494	hypothetical protein	YP_002329998.1	-2.67
Cluster_3612	E2348C_2894	hypothetical protein	YP_002330386.1	-2.73
Cluster_2670	E2348C_1809	ATPase, AAA-superfamily	YP_002329330.1	-2.86
Cluster_2879	E2348C_1234	ATP-binding protein	YP_002328782.1	-2.89
Cluster_5369	E2348C_2789	hypothetical protein	YP_002330287.1	-2.93
Cluster_7438	*rfaB*	UDP-D-galactose:(glucosyl)lipopolysaccharide-1,6-D-galactosyltransferase	YP_002331338.1	-2.96
Cluster_7204	E2348C_4647	helicase	YP_002332093.1	-3.04
Cluster_2584	E2348C_0834	hypothetical protein	YP_002328399.1	-3.08
Cluster_2898	E2348C_1839	hypothetical protein	YP_002329360.1	-3.10
Cluster_2113	E2348C_1099	hypothetical protein	YP_002328653.1	-3.15
Cluster_6478	E2348C_4589	hypothetical protein	YP_002332038.1	-3.19
Cluster_7030	E2348C_0982	hypothetical protein	YP_002328545.1	-3.26
Cluster_3756	E2348C_1100	hypothetical protein	YP_002328654.1	-3.28
Cluster_5693	E2348C_1098	hypothetical protein	YP_002328652.1	-3.30
Cluster_6306	E2348C_1401	hypothetical protein	YP_002328940.1	-3.50
Cluster_5251	E2348C_2099	DNA-binding protein	YP_002329613.1	-3.57
Cluster_5014	E2348C_2738	acyltransferase	YP_002330236.1	-3.58
Cluster_2891	*wbiO*	O-acetyltransferase	YP_002329686.1	-3.70
Cluster_4697	E2348C_0800	hypothetical protein	YP_002328366.1	-3.70
Cluster_5926	*wzx*	O-antigen flippase	YP_002329687.1	-3.75
Cluster_3167	E2348C_1232	hypothetical protein	YP_002328781.1	-3.79
Cluster_4162	E2348C_2111	hypothetical protein	YP_002329624.1	-3.85
Cluster_1491	E2348C_0799	hypothetical protein	YP_002328365.1	-3.92
Cluster_4023	E2348C_2535	hypothetical protein	YP_002330039.1	-4.18
Cluster_6252	E2348C_2112	hypothetical protein	YP_002329625.1	-4.21
Cluster_2060	E2348C_2493	hypothetical protein	YP_002329997.1	-4.21
Cluster_2140	E2348C_1400	hypothetical protein	YP_002328939.1	-4.29
Cluster_2499	E2348C_2105	hypothetical protein	YP_002329619.1	-4.34
Cluster_3539	*rfaZ*	lipopolysaccharide core biosynthesis protein	YP_002331334.1	-4.48
Cluster_4687	*wbiQ*	fucosyltransferase	YP_002329683.1	-4.56
Cluster_4553	E2348C_1074	hypothetical protein	YP_002328634.1	-4.64
Cluster_5439	E2348C_2920	hypothetical protein	YP_002330408.1	-4.78
Cluster_1608	E2348C_1235	HNH endonuclease	YP_002328783.1	-4.85
Cluster_3679	E2348C_0722	hypothetical protein	YP_002328289.1	-4.86
Cluster_2779	E2348C_2118	hypothetical protein	YP_002329630.1	-4.99
Cluster_5902	E2348C_2106	hypothetical protein	YP_002329620.1	-5.07
Cluster_6199	E2348C_2790	hypothetical protein	YP_002330288.1	-5.34
Cluster_2711	E2348C_2117	hypothetical protein	YP_002329629.1	-5.44
Cluster_7154	*wzy*	O-antigen polymerase	YP_002329685.1	-5.92
Cluster_4970	E2348C_2101	hypothetical protein	YP_002329615.1	-5.99
Cluster_4682	E2348C_2113	hypothetical protein	YP_002329626.1	-6.32
Cluster_4678	*rfaS*	lipopolysaccharide core biosynthesis protein	YP_002331339.1	-6.63
Cluster_3482	*wbiP*	glycosyl transferase family protein	YP_002329684.1	-6.81
C581-05				
Cluster_1384	None	conserved hypothetical protein	WP_000224835.1	3.28
Cluster_5749	None	putative aec2	WP_021528642.1	3.12
Cluster_6986	None	putative phage protein	WP_000206830.1	2.63
Cluster_7211	None	dnaJ domain protein	YP_002331854.1	2.59
Cluster_5894	None	carbohydrate binding domain protein	WP_024223345.1	2.17
Cluster_3621	None	DNA transfer protein gp7	WP_000964882.1	2.11
Cluster_5204	None	fimbrial family protein	WP_024235526.1	2.08
Cluster_4792	None	porin	WP_024234945.1	-2.01
Cluster_1359	None	polysaccharide biosynthesis family protein	WP_024235096.1	-2.05
Cluster_3884	None	hypothetical protein	None	-2.11
Cluster_2645	*rfaG*	glycosyl transferases group 1 family protein	WP_024235092.1	-2.14
Cluster_6444	None	hypothetical protein	WP_001276096.1	-2.14
Cluster_7160	None	phage integrase family protein	WP_024235378.1	-2.23
Cluster_6859	None	bacterial regulatory, Fis family protein	WP_000351362.1	-2.24
Cluster_7107	None	phosphoglucomutase/phosphomannomutase, C-terminal domain protein	WP_021569442.1	-2.24
Cluster_7182	None	phage integrase family protein	WP_024235494.1	-2.31
Cluster_7060	None	putative phage regulatory protein cI	WP_024235272.1	-2.34
Cluster_6342	*wekJ*	glycosyl transferase 2 family protein	ADB02824.1	-2.35
Cluster_3321	*argR*	arginine repressor, DNA binding domain protein	YP_002559154.1	-2.37
Cluster_6860	None	hypothetical protein	WP_024223156.1	-2.42

Cluster_2583	None	conserved hypothetical protein	WP_024234886.1	-2.44
Cluster_5915	None	kinase domain protein	WP_001022619.1	-2.49
Cluster_6127	None	conserved hypothetical protein	WP_024223157.1	-2.49
Cluster_5359	None	helix-turn-helix family protein	WP_001020632.1	-2.51
Cluster_6759	None	hypothetical protein	WP_024234795.1	-2.55
Cluster_6739	*yopT*-like	cysteine protease , YopT-type domain protein	WP_024235005.1	-2.75
Cluster_7387	None	formyl transferase family protein	WP_024233747.1	-2.84
Cluster_5941	None	putative membrane protein	WP_024235093.1	-2.86
Cluster_5617	None	conserved hypothetical protein	WP_001296199.1	-2.89
Cluster_4944	None	conserved hypothetical protein	WP_024235188.1	-2.93
Cluster_3633	None	putative putitive lysogenic protein	WP_024234808.1	-2.96
Cluster_2674	None	pyridoxal-5'-phosphate-dependent protein	WP_024235101.1	-3.00
Cluster_7279	None	conserved hypothetical protein	WP_024235280.1	-3.02
Cluster_7142	None	pfkB carbohydrate kinase family protein	WP_000667429.1	-3.15
Cluster_2400	None	hypothetical protein	WP_029789623.1	-3.25
Cluster_2672	None	hypothetical protein	WP_024234794.1	-3.51
Cluster_7459	None	hypothetical protein	WP_024235423.1	-3.53
Cluster_2401	None	UDP-glucose 4-epimerase	WP_021569447.1	-3.55
Cluster_5001	None	glycosyl transferases group 1 family protein	WP_021569444.1	-3.87
Cluster_6281	None	aminotransferase class-V family protein	WP_021569448.1	-3.89
Cluster_7343	None	conserved hypothetical protein	WP_024234803.1	-4.04
Cluster_6263	None	conserved hypothetical protein	WP_024235421.1	-4.10
Cluster_3642	None	conserved hypothetical protein	WP_001064017.1	-4.25
Cluster_6575	*grlR*	conserved hypothetical protein	WP_000581859.1	-4.59
Cluster_6916	*grlR*	conserved hypothetical protein	WP_023993845.1	-4.59
Cluster_5271	None	dTDP-Rha:alpha-D-GlcNAc-pyrophosphate polyprenol, alpha-3-L-rhamnosyltransferase	WP_024235099.1	-4.75
Cluster_4905	None	glycosyltransferase 9 family protein	WP_001100987.1	-4.85
Cluster_1900	None	putative membrane protein	WP_024234513.1	-4.91
Cluster_6114	None	TPR repeat family protein	WP_024235493.1	-4.91
Cluster_5231	*alaS*	hypothetical protein	WP_024235214.1	-5.59
B171				
Cluster_4761	*mhpR*	Mhp operon transcriptional activator	EDX27960.1	2.77
Cluster_4044	EcB171_2633	conserved hypothetical protein	EDX29304.1	2.68
Cluster_5485	*eamA*	eamA-like transporter family protein	WP_011251361.1	2.33
Cluster_5599	EcB171_5713	hypothetical protein	EDX27745.1	2.21
Cluster_6593	None	membrane protein	WP_012601962.1	-2.07
Cluster_5738	EcB171_0327	helix-turn-helix domain protein	EDX27874.1	-2.18
Cluster_4593	EcB171_0355	histidine kinase-, DNA gyrase B-, and HSP90-like ATPase family protein	EDX30341.1	-2.19
Cluster_6547	EcB171_0968	conserved hypothetical protein	EDX28365.1	-2.26
Cluster_3452	None	transcriptional regulator, GntR family	EDX31174.1	-2.26
Cluster_4695	*rdgC*	exonuclease, RdgC family protein	WP_000775327.1	-2.51
Cluster_4262	EcB171_1103	recombinase family protein	EDX32240.1	-2.56
Cluster_6786	EcB171_0681	putative repressor	EDX31498.1	-2.61
Cluster_7407	EcB171_2807	glycosyl transferases group 1 family protein	EDX31599.1	-2.76
Cluster_1575	None	methyltransferase domain protein	WP_001139852.1	-2.77
Cluster_6666	EcB171_0328	putative transmembrane protein	EDX27863.1	-2.94
Cluster_4177	EcB171_0329	conserved hypothetical protein	EDX27862.1	-3.87
Cluster_3976	None	putative membrane protein	WP_000448925.1	-3.88
Cluster_2102	None	putative predicted protein	WP_001430098.1	-4.98
E110019				
Cluster_7037	EcE110019_0331	conserved hypothetical protein	EDV85369.1	4.55
Cluster_4135	EcE110019_3655,<br>EcE110019_2513	phage terminase, small subunit, P27 family	EDV85325.1,<br>EDV85484.1	4.15
Cluster_6429	EcE110019_2810	conserved hypothetical protein	EDV89461.1	4.09
Cluster_6607	EcE110019_4614	antitermination family protein	EDV90062.1	2.81
Cluster_7246	*iutA*	ferric aerobactin receptor IutA	EDV87696.1	2.56
Cluster_5971	*iucD*	L-lysine 6-monooxygenase	EDV87688.1	2.42
Cluster_5382	EcE110019_4341	Phage anti-repressor protein	EDV86634.1	2.3
Cluster_1593	*antB*	antA/AntB antirepressor family protein	EDV87532.1	2.22
Cluster_4532	EcE110019_4625	conserved hypothetical protein	EDV90233.1	2.1
Cluster_3808	EcE110019_1931	putative membrane protein	EDV87574.1	-2
Cluster_7206	EcE110019_4502	conserved hypothetical protein	EDV86430.1	-2.05
Cluster_6289	EcE110019_4438	glycosyl transferase	EDV86461.1	-2.07
Cluster_3456	EcE110019_2812	helix-turn-helix family protein	EDV89547.1	-2.18
Cluster_1633	EcE110019_2426	transglycosylase SLT domain protein	EDV85380.1	-2.2
Cluster_4838	EcE110019_4469	transposase IS66 family protein	EDV86477.1	-2.25
Cluster_5169	*terB*	*terB*	EDV86451.1	-2.28
Cluster_3685	None	conserved hypothetical protein	WP_000277680.1	-2.28
Cluster_3437	EcE110019_2464	conserved hypothetical protein	EDV85329.1	-2.32
Cluster_4733	EcE110019_4464	putative aTP/GTP-binding protein	EDV86468.1	-2.39
Cluster_6471	*terD*	tellurium resistance protein TerD	EDV86434.1	-2.42
Cluster_6345	*terA*	tellurium resistance family protein	EDV86455.1	-2.42
Cluster_3524	None	conserved hypothetical protein	WP_001039671.1	-2.45
Cluster_6882	EcE110019_3368	conserved hypothetical protein	EDV88178.1	-2.49
Cluster_6229	EcE110019_2366	CAAX protease self-immunity family protein	EDV87100.1	-2.64
Cluster_4631	None	outer membrane insertion C-terminal signal domain protein	WP_001518282.1	-2.65
Cluster_7144	*parA*	plasmid partition protein A	EDV86708.1	-2.66
Cluster_5018	*terC*	integral membrane , TerC family protein	EDV86462.1	-2.73
Cluster_1871	*ureA*	urease subunit gamma	EDV86432.1	-2.73
Cluster_5137	*terZ*	tellurium resistance protein TerZ	EDV86470.1	-2.83
Cluster_5095	None	tyrosine kinase family protein	WP_029788025.1	-2.92
Cluster_5839	EcE110019_3367	conserved hypothetical protein	EDV88124.1	-2.92
Cluster_7071	EcE110019_4718	transposase family protein	EDV90183.1	-3.14
Cluster_3178	None	AAA-like domain protein, partial	WP_001329501.1	-3.15
Cluster_5836	EcE110019_2427	trbC/VIRB2 family protein	EDV85381.1	-3.37
Cluster_6390	EcE110019_1636	POTRA domain, ShlB-type family protein	EDV87624.1	-3.45
Cluster_1967	None	hypothetical protein	WP_029702172.1	-3.63
Cluster_6309	EcE110019_4463	conserved hypothetical protein	EDV86446.1	-3.65
Cluster_3269	None	trbL/VirB6 plasmid conjugal transfer family protein	WP_000046889.1	-3.77
Cluster_6100	EcE110019_4492	sulfatase family protein	EDV86483.1	-3.82
Cluster_6708	None	conserved hypothetical protein, partial	WP_001387332.1	-3.82
Cluster_1750	EcE110019_2425	conserved hypothetical protein	EDV85382.1	-3.83
Cluster_4541	None	type IV secretory pathway, VirB3-like family protein, partial	WP_001420214.1	-3.96
Cluster_6155	EcE110019_0678	P-type conjugative transfer protein VirB9	EDV85405.1	-4.27
Cluster_1365	EcE110019_4444	hypothetical protein	EDV86463.1	-4.47
Cluster_2719	EcE110019_0679	bacterial conjugation TrbI-like family protein	EDV85402.1	-4.53
Cluster_2101	None	initiator Replication family protein, partial	WP_000243324.1	-4.81
Cluster_6075	EcE110019_1635	AAA domain protein	EDV87677.1	-4.94
Cluster_6662	None	conserved hypothetical protein	WP_000970452.1	-5.23
Cluster_2766	EcE110019_2537	type IV secretion system s family protein	EDV85330.1	-5.64

[a]The gene symbol or locus id and the protein accession number are indicated for the top match protein. In some cases a protein match could not be identifed for a cluster in a particular genome, which likely results from differences in the gene-calling that was used for LS-BSR compared to that used for the GenBank sequences. None indicates there was not a corresponding locus id for the particular genome.
